# Associations between red meat, processed red meat and total red and processed red meat consumption, nutritional adequacy and markers of health and cardio-metabolic diseases in British adults: a cross-sectional analysis using data from UK National Diet and Nutrition Survey

**DOI:** 10.1007/s00394-021-02486-3

**Published:** 2021-02-07

**Authors:** D. A. Hobbs-Grimmer, D. I. Givens, J. A. Lovegrove

**Affiliations:** 1 Department of Food and Nutritional Sciences, Hugh Sinclair Unit of Human Nutrition, University of Reading, Reading, RG6 6AP UK; 2 Institute for Cardiovascular and Metabolic Research, University of Reading, Reading, UK; 3 Institute for Food, Nutrition and Health, University of Reading, Reading, UK

**Keywords:** Cardiometabolic health, Nutrient intakes, Red meat, Processed red meat

## Abstract

**Purpose:**

To determine the association between red meat (RM), processed red meat (PRM) and total red and processed red meat (TRPRM) consumption on nutritional adequacy and markers of health and cardio-metabolic diseases in British adults.

**Methods:**

In this cross-sectional study of adults (19–64 y) from the National Diet and Nutrition Survey (NDNS) (*n* = 1758), RM and PRM consumption were assessed from 4 day estimated food diaries. Anthropometric measures, blood pressure (BP), pulse pressure (PP), plasma glucose, HbA1c, C-reactive protein, TAG, TC, LDL-C and HDL-C from the NDNS were used.

**Results:**

43% of adults (men 57% and women 31%) consumed more than the 70 g/d TRPRM guidelines. Fewer adults in the highest tertile of TRPRM intake were below lower reference nutrient intakes (LRNIs), particularly for zinc and iron, respectively. In model 3 (controlled for age, energy intake, socioeconomic classification, number of daily cigarettes, BMI, dietary factors), higher RM consumption was associated with being significantly taller (model 3: *P-*ANCOVA = 0.006; *P*-T3/T1 = 0.0004) in men and lower diastolic BP (model 3: *P-*ANCOVA = 0.004; *P*-T3/T2 = 0.002) in women. Higher PRM in men was associated with significantly higher plasma ferritin concentration (model 3: *P-*ANCOVA = 0.0001; *P*-T2/T1 = 0.0001), being taller (*P-*ANCOVA = 0.019; *P*-T1/T2 = 0.047, T1/T3 = 0.044), increased body weight (model 3: *P-*ANCOVA = 0.001; *P*-T1/T3 = 0.0001), BMI (model 3: *P-*ANCOVA = 0.007; *P*-T1/T3 = 0.006) and smaller hip circumference (model 3: *P-*ANCOVA = 0.006; *P*-T3/T1 = 0.024; *P*-T2/T1 = 0.013) and in women significantly higher TC (model 3: *P-*ANCOVA = 0.020; *P*-T3/T2 = 0.016), LDL-C (*P-*ANCOVA = 0.030; *P*-T3/T2 = 0.025), HbA1c (model 3: *P-*ANCOVA = 0.0001; *P*-T2/T1 = 0.001; *P*-T3/T2 = 0.001) and higher PP (model 3: *P-*ANCOVA = 0.022; *P*-T3/T1 = 0.021).

Higher PRM consumption was associated with significantly higher BMI and hip circumference in men, and higher TC, LDL-C, HbA1c and PP in women, which was not observed for RM consumption.

**Supplementary Information:**

The online version contains supplementary material available at 10.1007/s00394-021-02486-3.

## Introduction

UK public health guidelines recommend that individuals eating more than 90 g of total red and processed meat per day should reduce this to 70 g per day [1]. This is largely based on the World Cancer Research Fund (2018) report, which concluded that the evidence was ‘convincing’ that red meat (RM) and processed meat were causes of colorectal cancer [2]. This was later supported by The International Agency for Research on Cancer (IARC), who classified processed meat as ‘carcinogenic to humans’ and RM as ‘probably carcinogenic to humans [3]. Evidence from epidemiological studies suggest that individuals with higher intakes of RM and processed meat have a greater risk of developing type 2 diabetes mellitus [4, 5], cardiovascular disease (CVD) [6] and certain cancers [7, 8]. However, findings from randomized controlled trials (RCTs) assessing the effect of RM consumption on CVD risk factors are inconsistent [9–11].

A meta-analysis of 24 RCTs assessing the effects of consuming ≥ 0.5 or < 0.5 servings of total red meat per day on CVD risk factors showed that the consumption of ≥ 0.5 servings of total red meat per day did not influence blood lipids and lipoproteins or blood pressures in comparison with < 0.5 servings per day [9]. An updated meta-analysis of 36 RCTs showed that high-quality plant protein resulted in more favorable changes in total and low-density lipoprotein (LDL) cholesterol in comparison with red meat intake, when changes in CVD risk factors was stratified by the specific foods used in the comparison/control diet [11].

Moreover, RM is a good source of a number of micro-nutrients in the diet, particularly iron and zinc, and it has been reported that diets containing less than 40 g RM per day may have implications for intakes of these micro-nutrients, particularly in women who have the lowest habitual intakes of unprocessed RM [12].

According to unweighted data from the most recent NDNS years 7 and 8 (combined), the current average total red and processed meat intakes for UK adults aged 19–64 years old are 60 g per day (76 g/d for men and 48 g/d for women) [13], which are substantially lower compared to 72 g/d (89 g/d for men and 56 g/d for women), reported in years 1–2 (combined) of the NDNS [14]. To date, few studies have evaluated differences in nutrient intakes and adequacy between diets containing varying levels of RM, processed red meat (PRM) and total red and processed red meat (TRPRM) intakes in the UK adult population, along with associations with health markers and risk factors for cardio-metabolic disease. The aim of the current research was to determine nutrient intakes and adequacy of diets containing varying levels of RM, PRM and TRPRM, and associations with health markers and risk factors for cardio-metabolic disease using cross-sectional data from years 1 to 4 of the NDNS.

## Methods

### Study population

The NDNS is a cross-sectional survey of the food consumption, nutritional intakes and nutritional status of a randomly selected demographically representative sample, comprising 2697 individuals (men *n* = 1126 and women *n* = 1571) living in private households across the UK between 2008 and 2012 [15]. A detailed description of the recruitment and study protocol have been reported previously [15]. Individuals with fasted blood glucose levels above 7 mmol/L or taking medicines known to affect blood analytes were excluded from the present analysis (*n* = 939). The final sample size was 1758 (men *n* = 801 and women *n* = 957). Due to a large number of missing blood samples (*n* = 1175) and anthropometric data (*n* = 747), the final participant numbers were further reduced to *n* = 583 (men *n* = 270 and women *n* = 313) and *n* = 1011 (men *n* = 492 and women *n* = 519) in the blood analyte and anthropometric data analysis, respectively. The NDNS was conducted according to the guidelines laid down in the Declaration of Helsinki, and ethical approval for all procedures was granted by Local Research Ethics Committees covering all areas in the survey. All participants gave informed consent.

### Dietary intake

Participants were asked to complete a 4 day food diary, which was completed on four consecutive days. The start dates for the 4 day food diaries were randomized to get a representative sample of all days of the week. Nutrient intakes from the 4 day diet diaries were calculated using the NDNS databank, which is based on McCance and Widdowson’s *Composition of Foods* series and the FSA’s *Food Portion Size guides.* The nutrient intakes reported in this analysis come only from foods consumed and do not include nutrients from vitamin or mineral supplements. In addition, salt added during cooking or at the table by participants was not included in the survey.

Nutritional adequacy was determined by comparing estimated nutrient intakes with UK nutrient recommendations and calculating the proportion of the population whose intake were below the Lower Reference Nutrient Intake (LRNI). The LRNI was defined as the amount of a nutrient that is likely to meet the needs of 2.5% of the population [16]. If individuals consume less than the LRNI, they will likely be deficient in that nutrient [15]. In our analysis, the threshold for considering population level intervention was 5% [17].

### Estimation of RM, PRM and TRPRM intakes

RM, PRM and TRPRM intakes were calculated based on the average weight of RM and PRM consumed per day from the 4 day diet diary using disaggregated data. In this analysis, the categorization of RM and PRM was performed according to the definition used by the IARC, with minor modification. Briefly, RM was defined as all types of mammalian muscle meat, such as beef, veal, pork, lamb, mutton, horse, and goat and processed red meat as red meat that has been transformed through salting, curing, fermentation, smoking, or other processes to enhance flavour or improve preservation. Most PRM contain pork or beef, but processed meats may also contain other red meats, offal, poultry or meat by-products such as blood. In our analysis, the RM food group included beef, lamb, pork, burgers and kebabs and other red meat (such as rabbit and venison) and the PRM food group included bacon and ham, sausages and other processed meat (such as corned beef and salami). TRPRM refers to the sum of all RM and PRM. The main difference in the definition of RM and PRM between the IARC classification and the one in this paper is that we excluded processed offal, poultry and meat by-products such as blood. Table 1 shows a breakdown of the RM and PRM food groups.

**Table 1:** Red and processed red meat food group definitions

Main food group	Sub-food group	Foods^a^
Red meat		
	Beef	Minced beef
	Lamb	Minced lamb
	Pork	Pork loin chops
	Burgers	Burgers made with 100% beef
	Other red meat	Rabbit
		Venison
Processed red meat		
	Bacon and ham	Ham
	Sausages	Pork sausages
	Other processed red meat	Corned beef

^a^Highest frequency of foods consumed within sub-food group

### Anthropometric measures and blood pressure

Participants also completed a computer based personal interview, collecting information on dietary habits and life-style, and had their height and weight measured, from which BMI was calculated. In a follow-up household visit by a nurse, waist and hip circumference and blood pressure were measured.

### Biochemical measurements

During the nurse visit, a fasted blood sample was also taken and this was subsequently analyzed for a number of analytes, including total serum total cholesterol (TC), high-density lipoprotein cholesterol (HDL-C), triacylglycerol (TAGs), C-reactive protein, homocysteine, plasma ferritin, glycated haemoglobin (HbA1c) and fasted glucose. The assays used for measurement of each analyte has been published previously [18]. Low-density lipoprotein cholesterol (LDL-C) was calculated using the Friedewald equation [19]. There was a 2–4-month period between dietary assessment and nurse visit [15].

### Data and statistical analysis

Complete case analysis was used. Variables were checked for normality by inspecting frequency distribution histograms, skew and kurtosis values. Data were organized into tertiles of RM, PRM and TRPRM consumption, with tertile 1 (T1) being the lowest consumers and tertile 3 (T3) being the highest consumers. Cardio-metabolic health markers, anthropometrics and nutrient intakes were treated as continuous variables when evaluating associations with meat tertiles. Analysis of covariance (ANCOVA) were used to test for associations between tertiles of RM, PRM and TRPRM consumption (independent variables) and nutrient intakes (dependent variables), cardio-metabolic health markers (dependent variables), anthropometric measures and blood pressure (dependent variables). The analyses testing the associations between RM, PRM and TRPRM tertiles and nutrient intakes were adjusted for age (continuous), total energy intake, socioeconomic classification (SEC) (categorical) and number of daily cigarettes (continuous). The analyses testing associations between cardio-metabolic health markers, anthropometric measures and blood pressure and RM, PRM and TRPRM tertiles were adjusted in base model for age (continuous), total energy intake, socioeconomic classification (SEC) (categorical) and number of daily cigarettes (continuous). To examine the effect independent of BMI analyses were further adjusted for BMI (continuous) (+ BMI). Further adjustment was made for fruit and vegetables, fibre, dairy, oily fish and nuts (+ dietary factors). Covariates were identified if known to be related based on previous published studies. We have also performed the statistical analysis using the residual method of energy adjustment and this did not change the results of the analysis. Bonferroni post hoc pairwise comparisons was used to determine differences between nutrient intakes and cardio-metabolic health markers across RM and PRM tertiles. Sensitivity analysis was conducted by performing the statistical analyses using quintiles and this did not change the results.

Differences in sociodemographic characteristics across tertiles of TRPRM consumption were determined using Chi-square test for independence. Data were weighted to account for non-response and sampling bias and this method has been described in detail elsewhere [20]. Briefly, the weighting factor corrected for known socioeconomic differences between the composition of the survey population and that of the total UK population, in terms of age by sex and Government Office Region. All statistical analysis was performed in SPSS for Windows 24 (SPSS Inc., Chicago, IL, USA). *P* values of < 0.05 were considered statistically significant.

## Results

The average TRPRM, RM and PRM intakes for adults aged 19–64 years old were 71.5 g/day (men 84.1 and women 55.8 g/day), 36.7 g/day (men 41.5 and women 30.8 g/day) and 34.7 g/day (men 42.6 and women 25.0 g/day), respectively. The percentage of adults exceeding the 70 g per day TRPRM guidelines was 43% (men 57% and women 31%).

Sociodemographic and health characteristics are presented in Table 2. Adults with the highest (T3) intakes of TRPRM were more likely to be a man, white, a current smoker and less educated compared with adults with the lowest (T1) TRPRM intakes (all *P* < 0.0001 apart from Socio-Economic Classification *P* = 0.016).

**Table 2:** Sociodemographic and health characteristics of British adults, by tertile of total red meat and processed red meat (TRPRM) intake^a^

	Total *n* = 1758	Tertiles of total red and processed red meat (TRPRM) (g/d)			
		1 (0–39) *n* = 582	2 (40–85) *n* = 593	3 (86–344) *n* = 583	*P*
Total red and processed red meat, g/d	71.5 ± 71.4	15.6 ± 17.7	61.6 ± 17.7	132.9 ± 57.7	0.0001
Red meat, g/d	36.8 ± 48.8	7.9 ± 14.3	32.1 ± 27.4	68.0 ± 56.8	0.0001
Processed red meat, g/d	34.8 ± 48.9	7.7 ± 13.0	29.6 ± 26.3	64.9 ± 60.2	0.0001
Total red and processed meat < or = 70 g/d, %	57	100	74	0	
Age, y	40.8 ± 12.1	40.7 ± 12.2	41.1 ± 12.4	40.6 ± 11.7	0.789
Men, %	46	32	39	65	0.0001
Qualifications, %					0.0001
Degree or equivalent	27	34	24	22	
Higher education, below degree level	12	12	14	10	
GCE, A level or equivalent	18	13	20	20	
GCSE grades A–C or equivalent	19	17	19	22	
GCSE grades D–G/Commercial qualifications/apprenticeship/foreign/other qualifications	5	6	5	7	
No qualifications/no response/still in education	19	18	19	19	
Equalized annual household income, £^b^	28,880 ± 25,214	28,880 ± 27,489	28,671 ± 24,170	29, 070 ± 23, 898	0.964
Socio-economic classification, %^c^					0.016
Higher managerial and professional occupations	16	20	16	14	
Lower managerial and professional occupations	25	24	28	24	
Intermediate occupations	10	9	11	9	
Small employers and own account workers	11	11	9	13	
Lower supervisory and technical occupations	9	7	8	12	
Semi-routine occupations	13	13	12	13	
Routine occupations	11	10	11	12	
Never worked/other	4	6	4	3	
Ethnic group, %					0.0001
White	92	88	93	94	
Any other group	9	13	7	6	
Number of daily cigarettes, %					0.0001
Non-smoker	54	61	57	46	
Ex-smoker	18	17	17	19	
Current smoker	28	22	26	35	
Has longstanding illness, % yes	20	21	19	19	0.779

^a^Values are means ± SD or percentages unless otherwise stated. NS, not significant. Differences between total red and processed red meat tertiles (TRPRM) for continuous variables were assessed using ANOVA and for categorical variables Chi-square test for independence was used

^b^The calculation of the equivalised income involves calculating a McClement score for each household (dependent on number, age and relationships of adults and children in the household), and then dividing the total household income by this score to get an equivalised household income

^c^Based on national statistics socioeconomic classification [40]

### Nutrient intakes and adequacy

#### Associations across RM tertiles

For both men and women total energy (MJ), protein, fat (% food energy), saturated fat (SFA, % food energy), MUFA (% food energy), trans fat (TFA, % food energy), niacin equivalents, vitamin B6, haem iron and zinc intakes were significantly higher and carbohydrate (% food energy), total sugars (% food energy) and calcium intakes were significantly lower with increasing tertiles of RM intake (Table 3). Vitamin B12, iron and selenium intakes for men and thiamin intakes for women were significantly higher across increasing RM tertiles. Cis n-6 fatty acids (% food intake) and iodine intakes for men and cis n-3 fatty acids (% food energy), fibre, folate and magnesium intakes for women were significantly lower across increasing RM tertiles (Table 3).

**Table 3:** Multivariate adjusted daily intakes of macro- and micro-nutrients and percentage of men and women aged 19–64 y below the Lower recommended Nutrient Intakes (LRNI) across red meat (RM) tertiles

Participants (*n*)	Tertiles of red meat (RM) consumption, g/d							
	Men				Women			
	T1	T2	T3	ANCOVA	T1	T2	T3	ANCOVA
	(0–13)	(14–60)	(61–224)	*P* value^1^	(0–8)	(9–35)	(36–233)	*P *value^1^
	265	266	270		319	320	318	
Total energy (MJ)	8.4 (8.1, 8.7)^a^	9.0 (8.7, 9.3)^b^	9.5 (9.2, 9.8)^b^	0.0001	6.3 (6.1, 6.5)^a^	6.7 (6.5, 6.9)^b^	7.0 (6.8, 7.2)^c^	0.0001
Protein (g)	81 (78, 84)^a^	83 (80, 86)^a^	94 (91, 97)^b^	0.0001	60 (59, 62)^a^	64 (63, 65)^b^	71 (70, 73)^c^	0.0001
Fat (g)	78 (76, 80)^a^	79 (77, 81)^a^	79 (77, 81)^a^	0.55	58 (57, 59)^a^	59 (58, 61)^a^	60 (59, 61)^a^	0.085
% food energy	34 (34, 35)^a^	35 (34, 36)^a^	36 (35, 36)^a^	0.039	33 (33, 34)^a^	34 (34, 35)^b^	35 (34, 35)^b^	0.022
SFA (g)	28 (27, 29)^a^	29 (29, 30)^b^	29 (28, 30)^ab^	0.044	21 (20, 21)^a^	22 (21, 23)^b^	22 (21, 22)^b^	0.011
% food energy	12 (12, 12)^a^	13 (13, 13)^b^	13 (13, 13)^b^	0.002	12 (12, 12)^a^	13 (12, 13)^b^	13 (12, 13)^b^	0.004
Cis MUFA (g)	28 (28, 29)^a^	29 (28, 30)^a^	29 (29, 30)^a^	0.28	21 (20, 21)^a^	21 (21, 22)^ab^	22 (21, 22)^b^	0.016
% food energy	13 (12, 13)^a^	13 (12, 13)^ab^	13 (13, 13)^b^	0.019	12 (12, 12)^a^	12 (12, 12)^a^	13 (12, 13)^b^	0.003
Cis n-6 FAs (g)	12 (12, 13)^a^	11 (11, 12)^b^	11 (11, 12)^b^	0.002	8.9 (8.5, 9.2)^a^	8.6 (8.3, 9)^a^	8.6 (8.3, 9.0)^a^	0.6
% food energy	5.5 (5.3, 5.7)^a^	5.0 (4.8, 5.2)^b^	5.1 (4.9, 5.3)^b^	0.001	5.1 (4.9, 5.3)^a^	5.0 (4.8, 5.2)^a^	5.0 (4.9, 5.2)^a^	0.74
Cis n-3 FAs (g)	2.3 (2.1, 2.4)^a^	2.1 (2.0, 2.3)^a^	2.2 (2.1, 2.3)^a^	0.40	2.0 (1.9, 2.1)^a^	1.8 (1.7, 1.9)^b^	1.6 (1.6, 1.7)^c^	0.0001
% food energy	1.0 (0.97, 1.1)^a^	0.94 (0.89, 0.99)^b^	1.0 (0.95, 1.1)^a^	0.062	1.1 (1.1, 1.2)^a^	1.0 (0.98, 1.1)^b^	0.97 (0.92, 1.0)^b^	0.0001
Trans fatty acids (g)	1.3 (1.2, 1.4)^a^	1.5 (1.4, 1.6)^b^	1.6 (1.5, 1.7)^b^	0.0001	1.0 (0.95, 1.1)^a^	1.1 (1.0, 1.2)^b^	1.2 (1.2, 1.3)^c^	0.0001
% food energy	0.58 (0.54, 0.61)^a^	0.67 (0.63, 0.70)^b^	0.72 (0.68, 0.75)^b^	0.0001	0.59 (0.55, 0.62)^a^	0.64 (0.61, 0.67)^a^	0.70 (0.68, 0.73)^b^	0.0001
Carbohydrate (g)	260 (255, 264)^a^	260 (255, 264)^a^	238 (234, 243)^b^	0.0001	204 (201, 207)^a^	196 (193, 199)^b^	187 (184, 190)^c^	0.0001
% food energy	49 (48, 50)^a^	48 (48, 49)^a^	45 (44, 46)^b^	0.0001	51 (50, 51)^a^	48 (48, 49)^b^	46 (45, 47)^c^	0.0001
Total sugars (g)	110 (106, 114)^a^	109 (105, 113)^a^	96 (92, 100)^b^	0.0001	84 (81, 87)^a^	86 (83, 89)^a^	77 (74, 80)^b^	0.0001
% food energy	21 (20, 21)^a^	20 (19, 21)^a^	18 (17, 19)^b^	0.0001	21 (20, 22)^a^	21 (20, 22)^a^	19 (18, 20)^b^	0.0001
Englyst fibre (g)	15 (15, 16)^a^	15 (14, 15)^a^	14 (14, 15)^a^	0.1	14 (13, 14)^a^	13 (12, 13)^b^	12 (12, 12)^c^	0.0001
Vitamin A (µg)^2^	902 (789, 1015)^a^	1049 (936, 1162)^a^	881 (771, 992)^a^	0.078	1003 (893, 1114)^a^	955 (848, 1063)^a^	941 (838, 1044)^a^	0.71
% below LRNI	12	9	10		7	7	5	
Thiamin (mg)	1.6 (1.5, 1.6)^a^	1.6 (1.5, 1.6)^a^	1.7 (1.6, 1.7)^b^	0.098	1.2 (1.2, 1.3)^a^	1.3 (1.2, 1.3)^b^	1.3 (1.3, 1.4)^b^	0.007
% below LRNI	0	0	0		0	0	0	
Riboflavin (mg)	1.8 (1.7, 1.8)^a^	1.7 (1.7, 1.8)^a^	1.7 (1.6, 1.8)^a^	0.68	1.4 (1.3, 1.4)^a^	1.4 (1.3, 1.4)^a^	1.3 (1.3, 1.4)^b^	0.71
% below LRNI	6	4	2		13	13	9	
Niacin eqv (mg)	42 (40, 43)^a^	41 (39, 42)^a^	46 (44, 48)^b^	0.0001	30 (29, 31)^a^	31 (31, 32)^a^	33 (32, 34)^b^	0.0001
% below LRNI	0	0	0		0	0	0	
Vitamin B6 (mg)	2.5 (2.4, 2.6)^a^	2.5 (2.4, 2.6)^a^	2.7 (2.6, 2.8)^a^	0.019	1.8 (1.7, 1.9)^a^	1.9 (1.8, 1.9)^ab^	2.0 (1.9, 2.0)^b^	0.04
% below LRNI	4	3	1		17	9	5	
Vitamin B12 (µg)	5.0 (4.6, 5.5)^a^	5.8 (5.3, 6.3)^b^	6.2 (5.8, 6.7)^b^	0.002	4.4 (4.1, 4.7)^a^	4.6 (4.3, 4.9)^a^	4.6 (4.3, 4.9)^a^	0.49
% below LRNI	2	0	0		4	1	0	
Folate (µg)	295 (283, 307)^a^	284 (272, 297)^a^	285 (273, 297)^a^	0.39	243 (235, 252)^a^	226 (218, 234)^b^	219 (212, 227)^b^	0.0001
% below LRNI	2	0	1		7	4	2	
Vitamin C (mg)	86 (79, 93)^a^	86 (79, 94)^a^	78 (71, 86)^a^	0.22	84 (77, 90)^a^	85 (79, 91)^a^	78 (72, 84)^a^	0.18
% below LRNI	3	0	0		1	1	1	
Sodium (mg)	2614 (2532, 2697)^a^	2671 (2589, 2754)^a^	2557 (2476, 2638)^a^	0.15	1994 (1936, 2052)^a^	2046 (1990, 2103)^a^	1961 (1907, 2015)^a^	0.098
% below LRNI	1	0	0		2	1	0	
Potassium (mg)	2985 (2912, 3058)^a^	3025 (2952, 3098)^a^	3081 (3009, 3153)^a^	0.19	2516 (2460, 2572)^a^	2468 (2413, 2523)^a^	2542 (2489, 2594)^a^	0.16
% below LRNI	13	13	6		30	27	14	
Calcium (mg)	915 (885, 945)^a^	910 (880, 940)^a^	841 (811, 870)^b^	0.001	737 (715, 760)^a^	715 (694, 737)^a^	679 (658, 699)^b^	0.001
% below LRNI	5	5	5		8	10	8	
Magnesium (mg)	288 (281, 295)^a^	277 (270, 284)^a^	282 (275, 289)^a^	0.11	232 (227, 237)^a^	221 (217, 226)^b^	218 (213, 222)^b^	0.0001
% below LRNI	17	17	11		15	15	9	
Iron (mg)	11 (11, 12)^a^	12 (11, 12)^a^	12 (12, 13)^b^	0.005	9.6 (9.3, 9.8)^ab^	9.4 (9.1, 9.6)^a^	9.8 (9.5, 10.0)^b^	0.070
% below LRNI	4	0	0		33	29	17	
Haem iron (mg)	0.52 (0.46, 0.57)^a^	0.78 (0.72, 0.83)^a^	1.1 (1.1, 1.2)^a^	0.0001	0.35 (0.32, 0.39)^a^	0.57 (0.53, 0.60)^b^	0.82 (0.79, 0.86)^c^	0.0001
Non-haem iron (mg)	11 (11, 11)^a^	11 (11, 11)^a^	11 (11, 12)^a^	0.58	9.2 (9.0, 9.4)^a^	8.8 (8.6, 9.0)^b^	8.9 (8.7, 9.1)^b^	0.059
Zinc (mg)	8.3 (8.1, 8.6)^a^	9.4 (9.1, 9.7)^b^	11 (11, 12)^c^	0.0001	6.4 (6.3, 6.6)^a^	7.3 (7.1, 7.5)^b^	8.8 (8.7, 9.0)^c^	0.0001
% below LRNI	19	5	0		12	3	0	
Iodine (µg)	189 (181, 197)^a^	179 (171, 187)^ab^	173 (165, 181)^b^	0.02	140 (135, 146)^a^	138 (132, 143)^a^	132 (127, 137)^a^	0.082
% below LRNI	6	6	3		12	10	9	
Selenium (µg)	54 (51, 56)^ab^	52 (49, 54)^a^	57 (54, 59)^b^	0.033	43 (42, 45)^a^	44 (42, 46)^a^	43 (41, 44)^a^	0.70
% below LRNI	35	27	16		57	52	50	

Values are multivariate adjusted means (95% CIs) or percentages unless otherwise stated

^1^Significant differences between red meat (RM) tertiles were determined by ANCOVA controlling for age, energy intake, socioeconomic classification (SEC) and number of daily cigarettes. Tertiles that do not share a superscripts letter were significantly different at *P*<0.05  based on Bonferroni post hoc pairwise comparisons

^2^Retinol equivalents

The percentage of men and women below the LRNI were less in the high (T3) compared with the low (T1) tertile of RM intake for the majority of nutrients (Table 3). For men, the biggest differences in the percentage of people below the LRNI between RM tertiles were seen for zinc (0% in T3 compared with 19% in T1) and selenium (16% in T3 compared with 35% in T1). For women, the biggest differences in the percentage of people below the LRNI between RM tertiles were seen for iron (17% in T3 compared with 33% in T1) and potassium (14% in T3 compared with 30% in T1).

#### Associations across PRM tertiles

For both men and women total energy (MJ), fat (% food energy), saturated fat (SFA, % food energy), MUFA (% food energy), thiamin, sodium, haem iron and non haem iron intakes were significantly higher and carbohydrate (% food energy), total sugars (% food energy), fibre, magnesium and selenium intakes were significantly lower with increasing tertiles of PRM intake (Table 4). Protein, riboflavin and zinc intakes were significantly higher for women across increasing PRM tertiles. Vitamins A and C for men and cis n-3 fatty acids, vitamin B12, folate and iodine intakes for women were significantly lower across increasing PRM tertiles (Table 4).

**Table 4:** Multivariate adjusted daily intakes of macro- and micro-nutrients and percentage of men and women aged 19–64 y below the Lower recommended Nutrient Intakes (LRNI) across processed red meat (PRM) tertiles

Participants (*n*)	Tertiles of processed red meat (PRM) consumption, g/d							
	Men				Women			
	T1	T2	T3	ANCOVA	T1	T2	T3	ANCOVA
	(0–20)	(21–57)	(58–284)	*P* value^1^	(0–7)	(8–30)	(31–162)	*P* value^1^
	272	263	266		313	320	324	
Total energy (MJ)	8.2 (7.9, 8.5)^a^	9.0 (8.7, 9.3)^b^	9.8 (9.5, 10.2)^c^	0.0001	6.3 (6.0, 6.5)^a^	6.7 (6.5, 6.9)^b^	7.2 (7.0, 7.4)^c^	0.0001
Protein (g)	87 (84, 90)^a^	85 (82, 88)^a^	87 (84, 90)^a^	0.72	64 (63, 65)^a^	64 (63, 65)^a^	69 (67, 70)^b^	0.0001
Fat (g)	77 (76, 79)^a^	78 (76, 80)^a^	82 (80, 84)^b^	0.001	59 (58, 60)^a^	58 (57, 59)^a^	61 (60, 62)^b^	0.002
% food energy	34 (33, 35)^a^	35 (34, 36)^b^	36 (35, 37)^b^	0.0001	34 (33, 34)^a^	34 (33, 34)^a^	35 (35, 36)^b^	0.0001
SFA (g)	28 (27, 28)^a^	29 (28, 30)^b^	30 (29, 31)^b^	0.004	21 (20, 21)^a^	21 (21, 22)^a^	23 (22, 23)^b^	0.0001
% food energy	12 (12, 12)^a^	13 (13, 13)^b^	13 (13, 13)^b^	0.0001	12 (11, 12)^a^	12 (12, 13)^b^	13 (13, 14)^c^	0.0001
Cis MUFA(g)	28 (27, 29)^a^	28 (28, 29)^a^	30 (30, 31)^b^	0.0001	21 (21, 22)^a^	21 (20, 21)^a^	22 (21, 22)^a^	0.030
% food energy	12 (12, 13)^a^	13 (13, 13)^b^	13 (13, 14)^b^	0.0001	12 (12, 12)^a^	12 (12, 13)^a^	13 (12, 13)^b^	0.003
Cis n-6 FAs (g)	12 (11, 12)^a^	11 (10, 11)^b^	12 (12, 13)^a^	0.0001	9.2 (8.9, 9.6)^a^	8.3 (8.0, 8.6)^b^	8.6 (8.3, 8.9)^b^	0.0001
% food energy	5.3 (5.1, 5.5)^a^	4.9 (4.7, 5.1)^b^	5.4 (5.2, 5.6)^a^	0.001	5.3 (5.1, 5.4)^a^	4.9 (4.7, 5.1)^b^	5.0 (4.9, 5.2)^b^	0.010
Cis n-3 FAs (g)	2.2 (2.1, 2.4)^a^	2.2 (2.1, 2.3)^a^	2.2 (2.0, 2.3)^a^	0.52	2.0 (1.9, 2.1)^a^	1.7 (1.6, 1.8)^b^	1.7 (1.6, 1.8)^b^	0.0001
% food energy	1.0 (0.96, 1.1)^a^	0.99 (0.94, 1.0)^a^	0.96 (0.9, 1.0)^a^	0.50	1.1 (1.1, 1.2)^a^	0.99 (0.94, 1.0)^b^	0.99 (0.94, 1.0)^b^	0.0001
Trans fatty acids (g)	1.5 (1.4, 1.5)^a^	1.5 (1.4, 1.6)^a^	1.5 (1.4, 1.6)^a^	0.74	1.1 (1.0, 1.1)^a^	1.1 (1.1, 1.2)^a^	1.1 (1.1, 1.2)^a^	0.48
% food energy	0.64 (0.61, 0.67)^a^	0.67 (0.63, 0.70)^b^	0.66 (0.62, 0.70)^b^	0.60	0.62 (0.59, 0.65)^a^	0.65 (0.62, 0.68)^b^	0.66 (0.63, 0.69)^b^	0.24
Carbohydrate (g)	258 (253, 262)^a^	249 (244, 254)^b^	249 (244, 254)^b^	0.013	203 (199, 206)^a^	195 (192, 198)^b^	188 (184, 191)^c^	0.0001
% food energy	49 (48, 49)^a^	47 (46, 48)^b^	46 (45, 47)^c^	0.0001	50 (49, 50)^a^	49 (48, 50)^a^	46 (46, 47)^b^	0.0001
Total sugars (g)	106 (102, 110)^a^	108 (104, 112)^a^	100 (96, 105)^b^	0.05	82 (79, 85)^ab^	85 (82, 88)^a^	79 (76, 82)^b^	0.020
% food energy	20 (19, 21)^a^	20 (19, 21)^a^	18 (18, 19)^b^	0.006	20 (19, 21)^ab^	21 (20, 22)^a^	19 (19, 20)^b^	0.006
Englyst fibre (g)	15 (15, 16)^a^	14 (14, 15)^b^	14 (14, 15)^b^	0.001	13 (13, 14)^a^	12 (12, 13)^b^	12 (12, 13)^b^	0.0001
Vitamin A (µg)^2^	946 (840, 1051)^a^	1121 (1009, 1233)^b^	743 (621, 864)^c^	0.0001	1066 (960, 1172)^a^	896 (790, 1002)^a^	931 (822, 1039)^a^	0.065
% below LRNI	11	11	8		8	6	4	
Thiamin (mg)	1.5 (1.5, 1.6)^a^	1.6 (1.5, 1.7)^b^	1.7 (1.7, 1.8)^c^	0.0001	1.2 (1.2, 1.3)^a^	1.2 (1.2, 1.3)^a^	1.4 (1.3, 1.4)^b^	0.0001
% below LRNI	0	0	0		0	0	0	
Riboflavin (mg)	1.7 (1.6, 1.8)^a^	1.8 (1.7, 1.9)^a^	1.7 (1.6, 1.8)^a^	0.21	1.4 (1.3, 1.4)^a^	1.3 (1.2, 1.3)^b^	1.4 (1.3, 1.4)^a^	0.003
% below LRNI	5	3	14		4	14	8	
Niacin eqv (mg)	43 (41, 45)^a^	43 (42, 45)^a^	42 (40, 44)^a^	0.61	32 (31, 33)^a^	31 (30, 32)^a^	32 (31, 33)^a^	0.094
% below LRNI	0	0	0		0	0	0	
Vitamin B6 (mg)	2.5 (2.4, 2.6)^a^	2.7 (2.5, 2.8)^a^	2.6 (2.4, 2.7)^a^	0.066	1.9 (1.8, 2.0)^a^	1.9 (1.8, 1.9)^a^	1.9 (1.8, 2.0)^a^	0.59
% below LRNI	4	1	13		3	14	5	
Vitamin B12 (µg)	5.6 (5.2, 6.1)^a^	6.1 (5.6, 6.6)^a^	5.3 (4.8, 5.8)^a^	0.06	4.9 (4.6, 5.2)^a^	4.2 (3.9, 4.5)^b^	4.5 (4.2, 4.8)^ab^	0.011
% below LRNI	1	0	2		1	3	0	
Folate (µg)	285 (274, 297)^a^	290 (278, 303)^a^	289 (276, 303)^a^	0.82	237 (229, 245)^a^	218 (210, 226)^b^	231 (223, 240)^a^	0.004
% below LRNI	1	2	6		1	5	2	
Vitamin C (mg)	90 (83, 97)^a^	86 (79, 93)^a^	73 (65, 80)^b^	0.004	83 (77, 89)^a^	84 (78, 90)^a^	78 (72, 84)^a^	0.31
% below LRNI	1	2	0		0	1	1	
Sodium (mg)	2305 (2234, 2376)^a^	2591 (2516, 2667)^b^	3031 (2949, 3113)^c^	0.0001	1854 (1801, 1907)^a^	1933 (1880, 1986)^b^	2219 (2164, 2273)^c^	0.0001
% below LRNI	1	0	0		0	2	0	
Potassium (mg)	3046 (2977, 3115)^a^	3060 (2986, 3133)^a^	2980 (2901, 3060)^a^	0.32	2530 (2476, 2584)^a^	2478 (2424, 2532)^a^	2521 (2465, 2576)^a^	0.36
% below LRNI	14	7	28		10	25	19	
Calcium (mg)	889 (860, 917)^a^	899 (868, 929)^a^	875 (841, 908)^a^	0.58	716 (694, 737)^a^	701 (679, 722)^a^	711 (689, 733)^a^	0.60
% below LRNI	7	1	10		6	11	5	
Magnesium (mg)	294 (287, 301)^a^	283 (276, 290)^b^	267 (260, 275)^c^	0.0001	233 (228, 238)^a^	219 (214, 224)^b^	218 (213, 223)^b^	0.0001
% below LRNI	19	11	16		13	15	8	
Iron (mg)	12 (12, 12)^a^	12 (11, 12)^a^	12 (11, 12)^a^	0.12	9.8 (9.6, 10)^a^	9.4 (9.2, 9.7)^b^	9.5 (9.2, 9.7)^b^	0.072
% below LRNI	3	0	31		1	27	21	
Haem iron (mg)	0.68 (0.62, 0.74)^a^	0.83 (0.77, 0.89)^b^	0.96 (0.89, 1.0)^c^	0.0001	0.51 (0.47, 0.55)^a^	0.58 (0.54, 0.62)^b^	0.7 (0.65, 0.74)^c^	0.0001
Non-haem iron (mg)	11 (11, 12)^a^	11 (10, 11)^b^	11 (10, 1.0)^b^	0.007	9.3 (9.1, 9.5)^a^	8.8 (8.6, 9.1)^b^	8.8 (8.5, 9.0)^b^	0.005
Zinc (mg)	9.8 (9.5, 10)^a^	9.7 (9.4, 10)^a^	9.7 (9.3, 10)^a^	0.90	7.4 (7.2, 7.6)^a^	7.5 (7.3, 7.7)^a^	7.8 (7.6, 8.0)^b^	0.036
% below LRNI	12	5	6		7	9	1	
Iodine (µg)	179 (171, 186)^a^	183 (175, 191)^a^	178 (169, 187)^a^	0.63	144 (138, 149)^a^	135 (130, 140)^b^	130 (125, 136)^b^	0.003
% below LRNI	6	3	13		6	11	7	
Selenium (µg)	57 (55, 59)^a^	53 (50, 55)^b^	52 (50, 55)^b^	0.019	46 (45, 48)^a^	42 (40, 43)^b^	42 (40, 43)^b^	0.0001
% below LRNI	32	21	56		24	54	48	

Values are multivariate adjusted means (95% CIs) or percentages unless otherwise stated

^1^Significant differences between processed red meat (PRM) tertiles were determined by ANCOVA controlling for age, energy intake, socioeconomic classification (SEC) and number of daily cigarettes. Tertiles that do not share a superscripts letter were significantly different at *P*<0.05 based on Bonferroni post hoc pairwise comparisons

^2^Retinol equivalents

The percentage of men and women below the LRNI were generally higher in the higher (T3) compared with the lower (T1) tertile of PRM intake for the majority of nutrients (Table 4). For men, the biggest differences in the percentage of people below the LRNI between PRM tertiles were seen for iron (31% in T3 compared with 3% in T1) and selenium (56% in T3 compared with 32% in T1). For women, the biggest differences in the percentage of people below the LRNI between PRM tertiles were seen for selenium (48% in T3 compared with 24% in T1) and iron (21% in T3 compared with 1% in T1).

#### Associations across TRPRM tertiles

For both men and women, intakes of the majority of nutrients were higher with increasing tertile of TRPRM consumption and the proportion of people below the LRNI was also less with higher TRPRM intakes (Supplementary Table 1).

### Cardiometabolic risk markers

#### Associations across RM tertiles

In men, total cholesterol (total-C) to HDL-C ratio was significantly different across tertiles of RM consumption (ANCOVA: *P* = 0.048) when controlling for age, energy intake (kJ), socioeconomic classification and number of daily cigarettes (model 1, Table 5). This remained significant following additional adjustment for BMI (ANCOVA model 2: *P* = 0.027), with TC to HDL-C ratio being significantly lower in T3 compared with T1 (T3 vs. T1 model 2: *P* = 0.026, Table 5). However, after additional adjustment for dietary factors (model 3), the differences across TC to HDL-C ratio tertiles disappeared. For women, there were no significant differences in cardio-metabolic risk markers across RM tertiles (Table 5).

**Table 5:** Multivariable adjusted means for cardio-metabolic risk markers for men and women aged 19–64 y across red meat (RM) tertiles

Blood biomarkers	Tertiles of red meat (RM) consumption, g/d							
	Men				Women			
	T1 (0–13)	T2 (14–60)	T3 (61–157)	ANCOVA *P* value*	T1 (0–8)	T2 (9–35)	T3 (36–157)	ANCOVA *P* value*
Participants (*n*)	94	109	66		109	104	99	
Total-C (mmol/L)								
Base model	5.3 (5.1, 5.5)^a^	5.0 (4.8, 5.2)^a^	5.1 (4.8, 5.3)^a^	0.10	5.2 (5.0, 5.4)^a^	5.3 (5.1, 5.5)^a^	5.2 (5.1, 5.4)^a^	0.77
+ BMI	5.3 (5.1, 5.5)^a^	5.0 (4.8, 5.2)^a^	5.1 (4.8, 5.3)a	0.11	5.2 (5.0, 5.4)^a^	5.3 (5.1, 5.5)^a^	5.2 (5.0, 5.4)^a^	0.76
+ Dietary factors	5.3 (5.1, 5.5)^a^	5.0 (4.9, 5.2)^a^	5.0 (4.8, 5.3)a	0.085	5.2 (5.0, 5.4)^a^	5.3 (5.1, 5.5)^a^	5.2 (5.0, 5.4)^a^	0.75
HDL-C (mmol/L)								
Base model	1.3 (1.3, 1.4)^a^	1.3 (1.2, 1.4)^a^	1.4 (1.3, 1.5)^a^	0.20	1.6 (1.5, 1.7)^a^	1.7 (1.6, 1.8)^a^	1.6 (1.6, 1.7)^a^	0.37
+ BMI	1.3 (1.3, 1.4)^a^	1.3 (1.2, 1.4)^a^	1.4 (1.3, 1.5)^a^	0.079	1.6 (1.5, 1.7)^a^	1.7 (1.6, 1.7)^a^	1.6 (1.6, 1.7)^a^	0.28
+ Dietary factors	1.3 (1.3, 1.4)^a^	1.3 (1.2, 1.4)^a^	1.4 (1.3, 1.5)^a^	0.15	1.6 (1.5, 1.7)^a^	1.7 (1.6, 1.7)^a^	1.6 (1.6, 1.7)^a^	0.35
LDL-C (mmol/L)								
Base model	3.3 (3.2, 3.5)^a^	3.1 (3.0, 3.3)^a^	3.1 (2.9, 3.3)^a^	0.13	3.2 (3.0, 3.3)^a^	3.2 (3.0, 3.4)^a^	3.1 (3.0, 3.3)^a^	0.90
+ BMI	3.3 (3.1, 3.5)^a^	3.1 (3.0, 3.3)^a^	3.1 (2.9, 3.3)^a^	0.13	3.2 (3.0, 3.3)^a^	3.2 (3.0, 3.4)^a^	3.1 (3.0, 3.3)^a^	0.80
+ Dietary factors	3.3 (3.1, 3.5)^a^	3.1 (3.0, 3.3)^a^	3.1 (2.9, 3.3)^a^	0.16	3.2 (3.0, 3.3)^a^	3.2 (3.0, 3.4)^a^	3.1 (3.0, 3.3)^a^	0.79
Total:HDL-C ratio								
Base model	4.2 (4.0, 4.5)^a^	3.9 (3.7, 4.1)^a^	3.8 (3.5, 4.1)^a^	0.048	3.5 (3.3, 3.6)^a^	3.3 (3.1, 3.5)^a^	3.3 (3.1, 3.5)^a^	0.47
+ BMI	4.2 (4.0, 4.4)^a^	3.9 (3.7, 4.2)^ab^	3.7 (3.5, 4.0)^b^	0.027	3.5 (3.3, 3.7)^a^	3.3 (3.1, 3.5)^a^	3.3 (3.1, 3.4)^a^	0.30
+ Dietary factors	4.2 (4, 4.4)^a^	4.0 (3.7, 4.2)^a^	3.8 (3.5, 4.1)^a^	0.10	3.5 (3.3, 3.6)^a^	3.3 (3.1, 3.5)^a^	3.3 (3.1, 3.5)^a^	0.45
TAG (mmol/L)								
Base model	1.5 (1.3, 1.7)^a^	1.4 (1.2, 1.5)^a^	1.3 (1.1, 1.5)^a^	0.44	1.1 (1.0, 1.2)^a^	1.0 (0.9, 1.2)^a^	1.1 (1, 1.2)^a^	0.83
+ BMI	1.5 (1.3, 1.6)^a^	1.4 (1.2, 1.5)^a^	1.3 (1.1, 1.5)^a^	0.44	1.1 (1.0, 1.2)^a^	1.0 (0.9, 1.2)^a^	1.1 (1, 1.2)^a^	0.85
+ Dietary factors	1.5 (1.3, 1.6)^a^	1.4 (1.2, 1.5)^a^	1.3 (1.1, 1.5)^a^	0.37	1.1 (1.0, 1.2)^a^	1.0 (0.9, 1.2)^a^	1.1 (1, 1.2)^a^	0.86
Homocysteine (μmol/L)								
Base model	11 (9.9, 12)^a^	9.9 (8.7, 11)^a^	10 (8.7, 11)^a^	0.29	9.7 (9.0, 10)^a^	9.1 (8.3, 9.8)^a^	9.0 (8.3, 9.6)^a^	0.25
+ BMI	11 (9.9, 12)^a^	9.8 (8.7, 11)^a^	10 (8.7, 12)^a^	0.26	9.7 (9.0, 10)^a^	9.1 (8.3, 9.8)^a^	9.0 (8.3, 9.6)^a^	0.25
+ Dietary factors	11 (9.9, 12)^a^	9.9 (8.8, 11)^a^	10 (8.7, 12)^a^	0.31	9.9 (9.3, 11)^a^	9.1 (8.3, 9.8)^a^	8.8 (8.1, 9.4)^a^	0.057
CRP (mg/L)								
Base model	2.3 (1.8, 2.8)^a^	2.3 (1.8, 2.8)^a^	1.9 (1.3, 2.5)^a^	0.64	3.2 (2.1, 4.3)^a^	2.8 (1.6, 4.1)^a^	3.2 (2.2, 4.3)^a^	0.88
+ BMI	2.3 (1.8, 2.7)^a^	2.3 (1.9, 2.8)^a^	1.9 (1.3, 2.5)^a^	0.54	3.3 (2.2, 4.3)^a^	2.9 (1.7, 4.1)^a^	3.1 (2.1, 4.2)^a^	0.91
+ Dietary factors	2.3 (1.8, 2.8)^a^	2.3 (1.8, 2.8)^a^	1.9 (1.3, 2.5)^a^	0.65	3.3 (2.2, 4.4)^a^	2.9 (1.6, 4.1)^a^	3.1 (2.0, 4.1)^a^	0.87
Hb A1c (%)								
Base model	5.4 (5.3, 5.5)^a^	5.4 (5.3, 5.5)^a^	5.4 (5.3, 5.5)^a^	0.21	5.4 (5.3, 5.5)^a^	5.4 (5.3, 5.4)^a^	5.5 (5.4, 5.5)^a^	0.053
+ BMI	5.4 (5.3, 5.5)^a^	5.4 (5.3, 5.5)^a^	5.4 (5.3, 5.5)^a^	0.81	5.4 (5.4, 5.5)^a^	5.4 (5.3, 5.4)^a^	5.5 (5.4, 5.5)^a^	0.082
+ Dietary factors	5.4 (5.3, 5.5)^a^	5.4 (5.3, 5.5)^a^	5.4 (5.3, 5.5)^a^	0.94	5.4 (5.4, 5.5)^a^	5.4 (5.3, 5.4)^a^	5.5 (5.4, 5.5)^a^	0.16
Glucose (mmol/L)								
Base model	5.2 (5.1, 5.3)^a^	5.1 (4.9, 5.2)^a^	5.0 (4.8, 5.1)^a^	0.070	4.9 (4.8, 5.0)^a^	4.9 (4.8, 5.0)^a^	5.0 (4.9, 5.1)^a^	0.36
+ BMI	5.2 (5.1, 5.3)^a^	5.1 (5, 5.2)^a^	5.0 (4.8, 5.1)^a^	0.063	4.9 (4.8, 5.0)^a^	4.9 (4.8, 5.0)^a^	5.0 (4.9, 5.0)^a^	0.51
+ Dietary factors	5.2 (5.1, 5.3)^a^	5.1 (5, 5.2)^a^	5.0 (4.8, 5.1)^a^	0.070	4.9 (4.8, 5.0)^a^	4.9 (4.8, 5.0)^a^	5.0 (4.9, 5.0)^a^	0.67
Ferritin (μg/L)								
Base model	115 (97, 134)^a^	138 (121, 156)^a^	126 (104, 148)^a^	0.21	50 (40, 60)^a^	46 (35, 57)^a^	62 (52, 71)^a^	0.080
+ BMI	115 (97, 133)^a^	139 (122, 156)^a^	125 (103, 147)^a^	0.15	50 (41, 60)^a^	46 (35, 57)^a^	61 (52, 71)^a^	0.10
+ Dietary factors	119 (101, 137)^a^	140 (123, 157)^a^	118 (96, 140)^a^	0.16	51 (41, 61)^a^	46 (35, 57)^a^	61 (51, 71)^a^	0.14
Haemoglobin (g/L)								
Base model	149 (147, 151)^a^	148 (146, 149)^a^	151 (149, 153)^a^	0.12	131 (129, 133)^a^	131 (129, 133)^a^	133 (131, 135)^a^	0.23
+ BMI	149 (147, 151)^a^	148 (146, 150)^a^	151 (149, 153)^a^	0.15	131 (129, 133)^a^	131 (129, 133)^a^	133 (131, 134)^a^	0.28
+ Dietary factors	149 (147, 151)^a^	148 (146, 149)^a^	151 (149, 153)^a^	0.067	131 (129, 133)^a^	131 (129, 133)^a^	133 (131, 135)^a^	0.16

Values are multivariate adjusted means (95% CIs)

*Significant differences between means across red meat (RM) tertiles were determined by ANCOVA controlling for age, energy intake, socioeconomic classification (SEC) and number of daily cigarettes (base model), with additional adjustment for BMI (+ BMI) and further adjustment for fruit and vegetables, fibre, dairy, oily fish and nuts (+ dietary factors). Tertiles that do not share a superscripts letter were significantly different at *P*<0.05 based on Bonferroni post hoc pairwise comparisons

#### Associations across PRM tertiles

In men, triacylglycerol and homocysteine concentrations were significantly different across tertiles of PRM consumption (ANCOVA model 1: *P* = 0.009 and *P* = 0.032, respectively, Table 6). Following additional adjustment for BMI (model 2), triacylglycerol and homocysteine concentrations remained significantly different across tertiles of PRM consumption (ANCOVA model 2: *P* = 0.030 and *P* = 0.024, respectively) with triacylglycerol and homocysteine levels being significantly higher in T2 compared with T1 (T2 vs. T1 model 2: *P* = 0.028 and *P* = 0.020 for triacylglycerol and homocysteine, respectively). However, after additional adjustment for dietary factors (model 3), the differences across triacylglycerol and homocysteine tertiles disappeared. HbA1c concentration was also significantly different across PRM tertiles (ANCOVA model 2: *P* = 0.034 and model 3: *P* = 0.011), with HbA1c concentration being significantly lower in T2 compared with T1 (T2 vs. T1 model 2: *P* = 0.037 and model 3: *P* = 0.013, Table 6). Glucose concentration was significantly different across PRM tertiles, but only in model 1 (ANCOVA: *P* = 0.022), with glucose concentration being significantly higher in T3 compared with T1 (T3 vs. T1: *P* = 0.019, Table 6). Plasma ferritin concentration was significantly different across PRM tertiles in all models (ANCOVA model 1: *P* = 0.0001; model 2: *P* = 0.0001 and model 3: *P* = 0.0001) with concentrations being significantly higher in T2 compared with T1 (T2 vs. T1 model 1: *P* = 0.0001; model 2: *P* = 0.0001 and model 3: *P* = 0.0001, respectively).

**Table 6:** multivariable adjusted means for cardio-metabolic risk markers for men and women aged 19–64 y across processed red meat (PRM) tertiles

Blood biomarkers	Tertiles of processed red meat (PRM) consumption, g/d							
	Men				Women			
	T1 (0–20)	T2 (21–57)	T3 (58–165)	ANCOVA *P* value*	T1 (0–7)	T2 (8–30)	T3 (31–162)	ANCOVA *P* value*
Participants (*n*)	88	89	92		100	110	102	
Total-C (mmol/L)								
Base model	5.1 (4.9, 5.3)^a^	5.2 (5.0, 5.4)^a^	5.1 (4.8, 5.3)^a^	0.66	5.3 (5.1, 5.4)^ab^	5.1 (4.9, 5.2)^a^	5.4 (5.2, 5.6)^b^	0.027
+ BMI	5.1 (5.0, 5.3)^a^	5.2 (5.0, 5.4)^a^	5.0 (4.8, 5.2)^a^	0.42	5.3 (5.1, 5.5)^ab^	5.1 (4.9, 5.2)^a^	5.4 (5.2, 5.6)^b^	0.021
+ Dietary factors	5.2 (5.0, 5.4)^a^	5.2 (5.0, 5.3)^a^	5.0 (4.8, 5.2)^a^	0.45	5.2 (5.0, 5.4)^ab^	5.1 (4.9, 5.2)^a^	5.4 (5.2, 5.6)^b^	0.020
HDL-C (mmol/L)								
Base model	1.4 (1.3, 1.5)^a^	1.3 (1.3, 1.4)^a^	1.3 (1.2, 1.4)^a^	0.20	1.7 (1.6, 1.7)^a^	1.6 (1.5, 1.7)^a^	1.6 (1.5, 1.7)^a^	0.58
+ BMI	1.4 (1.3, 1.4)^a^	1.3 (1.3, 1.4)^a^	1.3 (1.3, 1.4)^a^	0.75	1.7 (1.6, 1.7)^a^	1.6 (1.5, 1.7)^a^	1.6 (1.5, 1.7)^a^	0.72
+ Dietary factors	1.4 (1.3, 1.4)^a^	1.3 (1.3, 1.4)^a^	1.3 (1.3, 1.4)^a^	0.87	1.6 (1.6, 1.7)^a^	1.6 (1.5, 1.7)^a^	1.6 (1.5, 1.7)^a^	0.88
LDL-C (mmol/L)								
Base model	3.2 (3.1, 3.4)^a^	3.2 (3.0, 3.3)^a^	3.1 (2.9, 3.3)^a^	0.69	3.1 (2.9, 3.3)^ab^	3.1 (2.9, 3.2)^a^	3.4 (3.2, 3.5)^b^	0.033
+ BMI	3.3 (3.1, 3.4)^a^	3.2 (3.0, 3.4)^a^	3.1 (2.9, 3.2)^a^	0.30	3.1 (3.0, 3.3)^ab^	3.0 (2.9, 3.2)^a^	3.4 (3.2, 3.5)^b^	0.025
+ Dietary factors	3.3 (3.1, 3.5)^a^	3.2 (3.0, 3.3)^a^	3.0 (2.9, 3.2)^a^	0.15	3.1 (3.0, 3.3)^ab^	3.0 (2.9, 3.2)^a^	3.3 (3.2, 3.5)^b^	0.030
Total:HDL-C ratio								
Base model	4.0 (3.7, 4.2)^a^	3.9 (3.7, 4.2)^a^	4.1 (3.8, 4.4)^a^	0.78	3.4 (3.2, 3.7)^a^	3.2 (3, 3.4)^a^	3.4 (3.2, 3.6)^a^	0.17
+ BMI	4.1 (3.8, 4.3)^a^	4.0 (3.7, 4.2)^a^	3.9 (3.7, 4.2)^a^	0.74	3.5 (3.3, 3.7)^a^	3.2 (3, 3.4)^a^	3.4 (3.2, 3.6)^a^	0.067
+ Dietary factors	4.1 (3.9, 4.3)^a^	4.0 (3.7, 4.2)^a^	3.9 (3.6, 4.1)^a^	0.38	3.5 (3.3, 3.7)^a^	3.2 (3, 3.4)^a^	3.4 (3.2, 3.6)^a^	0.12
TAG (mmol/L)								
Base model	1.2 (1.0, 1.4)^a^	1.5 (1.4, 1.7)^b^	1.5 (1.3, 1.7)^b^	0.009	1.1 (1.0, 1.3)^a^	1.0 (0.9, 1.1)^a^	1.1 (1.0, 1.2)^a^	0.17
+ BMI	1.2 (1.0, 1.4)^a^	1.5 (1.4, 1.7)^b^	1.5 (1.3, 1.6)^ab^	0.030	1.1 (1.0, 1.3)^a^	1.0 (0.9, 1.1)^a^	1.1 (1.0, 1.2)^a^	0.13
+ Dietary factors	1.3 (1.0.1, 1.4)^a^	1.5 (1.4, 1.7)^a^	1.4 (1.3, 1.6)^a^	0.054	1.1 (1.0, 1.2)^a^	1.0 (0.9, 1.1)^a^	1.1 (1.0, 1.2)^a^	0.41
Homocysteine (μmol/L)								
Base model	9.3 (8.1, 10)^a^	11 (10, 13)^b^	10 (9.0, 12)^a^	0.033	9.4 (8.7, 10)^a^	9.1 (8.5, 9.8)^a^	9.2 (8.5, 9.9)^a^	0.85
+ BMI	9.2 (8.0, 10)^a^	11 (10, 13)^b^	11 (9.2, 12)^ab^	0.024	9.4 (8.7, 10)^a^	9.1 (8.5, 9.8)^a^	9.2 (8.5, 10)^a^	0.85
+ Dietary factors	9.3 (8.1, 10)^a^	11 (10, 12)^a^	11 (9.2, 12)^a^	0.059	9.6 (8.9, 10)^a^	9.0 (8.3, 9.6)^a^	9.2 (8.5, 10)^a^	0.41
CRP (mg/L)								
Base model	2.0 (1.5, 2.5)^a^	2.3 (1.8, 2.8)^a^	2.3 (1.7, 2.8)^a^	0.74	3.5 (2.4, 4.7)a	2.5 (1.4, 3.5)a	3.5 (2.3, 4.6)a	0.32
+ BMI	2.1 (1.6, 2.6)^a^	2.3 (1.8, 2.8)^a^	2.1 (1.5, 2.7)^a^	0.81	3.6 (2.5, 4.8)a	2.4 (1.4, 3.4)a	3.5 (2.3, 4.6)a	0.21
+ Dietary factors	2.1 (1.6, 2.6)^a^	2.4 (1.9, 2.9)^a^	2.1 (1.5, 2.6)^a^	0.67	3.7 (2.5, 4.9)a	2.3 (1.3, 3.4)a	3.5 (2.3, 4.6)a	0.17
Hb A1c (%)								
Base model	5.5 (5.4, 5.5)^a^	5.3 (5.2, 5.4)^a^	5.4 (5.3, 5.5)^a^	0.066	5.5 (5.4, 5.5)^a^	5.3 (5.3, 5.4)^b^	5.5 (5.4, 5.6)^a^	0.001
+ BMI	5.5 (5.4, 5.6)^a^	5.3 (5.2, 5.4)^b^	5.4 (5.3, 5.5)^ab^	0.034	5.5 (5.4, 5.5)^a^	5.3 (5.3, 5.4)^b^	5.5 (5.4, 5.5)^a^	0.0001
+ Dietary factors	5.5 (5.4, 5.6)^a^	5.3 (5.2, 5.4)^b^	5.3 (5.2, 5.4)^ab^	0.011	5.5 (5.4, 5.5)^a^	5.3 (5.3, 5.4)^b^	5.5 (5.4, 5.5)^a^	0.0001
Glucose (mmol/L)								
Base model	5.0 (4.9, 5.1)^a^	5.1 (4.9, 5.2)^ab^	5.2 (5.1, 5.4)^b^	0.022	4.9 (4.8, 5.0)^a^	5.0 (4.9, 5.1)^a^	4.9 (4.8, 5.0)^a^	0.49
+ BMI	5.0 (4.9, 5.1)^a^	5.1 (5.0, 5.2)^a^	5.2 (5.1, 5.3)^a^	0.13	4.9 (4.8, 5.0)^a^	5.0 (4.9, 5.0)^a^	4.9 (4.8, 5.0)^a^	0.64
+ Dietary factors	5.0 (4.9, 5.1)^a^	5.1 (4.9, 5.2)^a^	5.2 (5.1, 5.3)^a^	0.23	4.9 (4.8, 5.0)^a^	5.0 (4.9, 5.0)^a^	4.9 (4.8, 5.0)^a^	0.55
Ferritin (μg/L)								
Base model	101 (84, 119)^a^	153 (135, 171)^b^	127 (106, 147)^ab^	0.0001	54 (44, 65)^a^	57 (48, 67)^a^	48 (38, 59)^a^	0.43
+ BMI	104 (87, 122)^a^	154 (136, 171)^b^	122 (101, 142)^ab^	0.0001	55 (44, 65)^a^	57 (48, 67)^a^	48 (38, 59)^a^	0.44
+ Dietary factors	99 (82, 117)^a^	154 (136, 171)^b^	128 (108, 149)^ab^	0.0001	57 (46, 67)^a^	57 (48, 67)^a^	47 (36, 57)^a^	0.29
Haemoglobin (g/L)								
Base model	148 (146, 150)^a^	149 (147, 151)^a^	150 (148, 152)^a^	0.27	130 (128, 131)^a^	133 (131, 134)^a^	132 (130, 134)^a^	0.048
+ BMI	148 (147, 150)^a^	149 (147, 151)^a^	150 (148, 152)^a^	0.43	130 (128, 132)^a^	133 (131, 134)^a^	132 (130, 134)^a^	0.062
+ Dietary factors	149 (147, 150)^a^	149 (147, 150)^a^	150 (148, 152)^a^	0.69	130 (128, 132)^a^	133 (131, 135)^a^	132 (130, 134)^a^	0.086

Values are multivariate adjusted means (95% CIs)

*Significant differences between means across processed red meat (PRM) tertiles were determined by ANCOVA controlling for age, energy intake, socioeconomic classification (SEC) and number of daily cigarettes (Base model), with additional adjustment for BMI (+ BMI) and further adjustment for fruit and vegetables, fibre, dairy, oily fish and nuts (+ dietary factors). Tertiles that do not share a superscripts letter were significantly different at *P*<0.05 based on Bonferroni post hoc pairwise comparisons

In women, TC and LDL-C concentrations were significantly different across tertiles of PRM consumption (ANCOVA model 1: *P* = 0.027 and *P* = 0.033; model 2: *P* = 0.021 and *P* = 0.025; model 3: *P* = 0.020 and *P* = 0.030 for total-C and LDL-C, respectively) with TC and LDL-C concentration being significantly higher in T3 compared with T2 (T3 Vs. T2 model 1: *P* = 0.023 and *P* = 0.035; model 2: *P* = 0.019 and *P* = 0.022; model 3: *P* = 0.016 and *P* = 0.025 for TC and LDL-C, respectively, Table 6). HbA1c concentration was significantly different across PRM tertiles in all models (ANCOVA model 1: *P* = 0.001; model 2: *P* = 0.0001 and model 3: *P* = 0.0001) with concentrations being significantly lower in T2 compared with T1 and T3 (T2 Vs. T1 model 1: *P* = 0.02; model 2: *P* = 0.007; model 3: *P* = 0.001 and T2 Vs. T3 model 1: *P* = 0.002; model 2: *P* = 0.001; model 3: *P* = 0.001). Haemoglobin levels were also significantly different across PRM tertiles (ANCOVA model 1: *P* = 0.048), but this disappeared after adjustment for BMI (model 2) and dietary factors (model 3).

### Anthropometrics and blood pressure

#### Associations across RM tertiles

In men, there was a significant difference in height across tertiles of RM consumption (ANCOVA model 1: *P* = 0.013 and model 3: *P* = 0.006) with men in the highest tertile (T3) of RM consumption being significantly taller than men in the lowest tertile (T1) of RM intake (T3 Vs. T1 model 1: *P* = 0.01 and model 3: T3 vs. T1 *P* = 0.004, Table 7). Pulse pressure (PP) was significantly different across RM tertiles in all models (ANCOVA model 1: *P* = 0.034; model 2: *P* = 0.036 and model 3: *P* = 0.040), but Bonferroni post hoc pairwise comparisons revealed that there were no significant differences between RM tertiles.

**Table 7:** Multivariate adjusted anthropometric measures and blood pressure for men and women aged 19–64 y across red meat (RM) tertiles

Biomarkers	Tertiles of red meat (RM) consumption, g/d							
	Men				Women			
	T1 (0–13)	T2 (14–60)	T3 (61–213)	ANCOVA *P* value*	T1 (0–8)	T2 (9–35)	T3 (36–158)	ANCOVA *P* value*
Participants (*n*)	166	197	127		186	164	169	
Height (cm)								
Base model	176 (175, 177)^a^	177 (176, 178)^ab^	178 (177, 179)^b^	0.013	162 (161, 163)^a^	163 (162, 164)^a^	163 (162, 164)^a^	0.47
+ BMI	–	–	–		–	–	–	
+ Dietary factors	176 (175, 177)^a^	177 (176, 178)^ab^	178 (177, 179)^b^	0.006	162 (161, 163)^a^	163 (162, 164)^a^	163 (162, 164)^a^	0.20
Weight (kg)								
Base model	83 (81, 85)^a^	84 (82, 86)^a^	85 (83, 87)^a^	0.40	70 (68, 72)^a^	71 (68, 73)^a^	71 (69, 73)^a^	0.68
+ BMI	–	–	–		–	–	–	
+ Dietary factors	83 (81, 85)^a^	84 (82, 86)^a^	85 (83, 88)^a^	0.28	70 (68, 72)^a^	71 (68, 73)^a^	71 (69, 73)^a^	0.71
BMI (kg/m^2^)								
Base model	27 (26, 27)^a^	27 (26, 27)^a^	27 (26, 27)^a^	0.95	27 (26, 27)^a^	27 (26, 28)^a^	27 (26, 28)^a^	0.84
+ BMI	–	–	–		–	–	–	
+ Dietary factors	27 (26, 27)^a^	27 (26, 28)^a^	27 (26, 28)^a^	0.90	27 (26, 28)^a^	27 (26, 28)^a^	27 (26, 28)^a^	0.91
Waist circumference (cm)								
Base model	94 (93, 96)^a^	95 (93, 96)^a^	95 (93, 97)^a^	0.84	86 (84, 88)^a^	86 (84, 88)^a^	87 (85, 89)^a^	0.57
+ BMI	94 (94, 95)^a^	95 (94, 95)^a^	95 (94, 96)^a^	0.63	86 (85, 87)^a^	86 (85, 87)^a^	87 (86, 88)^a^	0.50
+ Dietary factors	94 (94, 95)^a^	95 (94, 95)^a^	95 (94, 96)^a^	0.57	86 (85, 87)^a^	86 (85, 87)^a^	87 (86, 88)^a^	0.55
Hip circumference (cm)								
Base model	104 (103, 105)^a^	105 (104, 106)^a^	105 (103, 106)^a^	0.62	106 (104, 107)^a^	105 (103, 107)^a^	106 (104, 108)^a^	0.66
+ BMI	104 (104, 105)^a^	105 (104, 105)^a^	105 (104, 105)^a^	0.37	106 (105, 107)^a^	105 (104, 106)^a^	106 (105, 107)^a^	0.20
+ Dietary factors	104 (104, 105)^a^	105 (104, 105)^a^	105 (104, 106)^a^	0.19	106 (105, 106)^a^	105 (104, 106)^a^	106 (105, 107)^a^	0.27
Waist:hip ratio								
Base model	0.90 (0.89, 0.91)^a^	0.90 (0.90, 0.91)^a^	0.90 (0.89, 0.91)^a^	1.00	0.81 (0.80, 0.82)^a^	0.82 (0.81, 0.83)^a^	0.82 (0.81, 0.83)^a^	0.47
+ BMI	0.90 (0.90, 0.91)^a^	0.90 (0.90, 0.91)^a^	0.90 (0.90, 0.91)^a^	0.97	0.81 (0.80, 0.82)^a^	0.82 (0.81, 0.83)^a^	0.82 (0.81, 0.83)^a^	0.54
+ Dietary factors	0.90 (0.90, 0.91)^a^	0.90 (0.90, 0.91)^a^	0.90 (0.90, 0.91)^a^	0.92	0.82 (0.81, 0.83)^a^	0.82 (0.81, 0.83)^a^	0.82 (0.81, 0.83)^a^	0.98
SBP (mmHg)								
Base model	128 (126, 130)^a^	128 (126, 130)^a^	128 (126, 130)^a^	0.95	118 (116, 120)^a^	119 (117, 121)^a^	116 (114, 118)^a^	0.15
+ BMI	128 (126, 130)^a^	128 (126, 130)^a^	128 (126, 130)^a^	0.96	118 (116, 120)^a^	119 (117, 121)^a^	116 (114, 118)^a^	0.12
+ Dietary factors	128 (126, 129)^a^	128 (127, 130)^a^	128 (126, 131)^a^	0.76	118 (116, 120)^a^	119 (117, 121)^a^	116 (114, 118)^a^	0.11
DBP (mmHg)								
Base model	74 (72, 75)^a^	74 (72, 75)^a^	74 (72, 76)^a^	0.99	72 (70, 73)^ab^	74 (72, 76)^a^	70 (69, 72)^b^	0.005
+ BMI	74 (72, 75)^a^	74 (72, 75)^a^	74 (72, 76)^a^	0.98	72 (70, 73)^ab^	74 (72, 76)^a^	70 (69, 72)^b^	0.002
+ Dietary factors	74 (72, 75)^a^	74 (72, 75)^a^	74 (73, 76)^a^	0.77	72 (71, 73)^ab^	74 (72, 75)^a^	70 (69, 72)^b^	0.004
Pulse pressure (mmHg)								
Base model	67 (66, 69)^a^	69 (68, 71)^a^	67 (65, 69)^a^	0.034	72 (71, 73)^a^	73 (71, 74)^a^	71 (69, 72)^a^	0.17
+ BMI	67 (66, 69)^a^	69 (68, 71)^a^	67 (65, 69)^a^	0.036	72 (71, 73)^a^	73 (71, 74)^a^	71 (69, 72)^a^	0.17
+ Dietary factors	67 (66, 69)^a^	69 (68, 71)^a^	67 (65, 69)^a^	0.040	72 (70, 73)^a^	72 (71, 74)^a^	71 (69, 72)^a^	0.32

Values are multivariate adjusted means (95% CIs)

*Significant differences between means across red meat (RM) tertiles were determined by ANCOVA controlling for age, energy intake, socioeconomic classification (SEC) and number of daily cigarettes (Base model), with additional adjustment for BMI (+ BMI) and further adjustment for fruit and vegetables, fibre, dairy, oily fish and nuts (+ dietary factors). For height and weight model 3 did not include adjustment for BMI. Tertiles that do not share a superscripts letter were significantly different at *P*<0.05 based on Bonferroni post hoc pairwise comparisons

For women, there was a significant difference in diastolic blood pressure across tertiles of RM consumption in all models (ANCOVA model 1: *P* = 0.005; model 2: *P* = 0.002 and model 3: *P* = 0.004) with diastolic blood pressure being significantly lower in T3 compared with T2 (model 1: T3 vs. T2 *P* = 0.004; model 2: T3 vs. T2 *P* = 0.002 and model 3: T3 vs. T2 *P* = 0.002, Table 7).

#### Associations across PRM tertiles

In men, there was a significant difference in height, weight and BMI across tertiles of PRM consumption (ANCOVA model 1: *P* = 0.010; *P* = 0.0001 and *P* = 0.006, respectively). This remained significant following additional adjustment for dietary factors (ANCOVA model 3: *P* = 0.019, *P* = 0.0001 and *P* = 0.007 for height, weight and BMI, respectively) with men in the highest (T3) and middle (T2) tertiles being significantly taller than men in the lowest (T1) tertiles of PRM consumption (model 3: T3 vs. T1 *P* = 0.044 and T2 vs. T1 *P* = 0.047, Table 8). In addition, men in the highest (T3) tertile of PRM intake also weighed significantly more and had a higher BMI compared to men in the lowest (T1) tertiles of PRM intake (model 3: for weight T3 vs. T1 *P* = 0.0001 and BMI T3 vs. T1 *P* = 0.006). Waist circumference was also significantly different across PRM tertiles in model 1 (ANCOVA *P* = 0.002), but this disappeared with additional adjustment for BMI (model 2) and dietary factors (model 3). Hip circumference was significantly different across tertiles of PRM consumption in all models (ANCOVA model 1: *P* = 0.0001 model 2: *P* = 0.004 and model 3: *P* = 0.006) with men in the highest (T3) and middle (T2) tertiles of PRM consumption having significantly smaller hip circumference compared with men in T1 (model 3: T2 vs. T1 *P* = 0.013) and T3 (model 3: T3 vs. T1 *P* = 0.024, Table 8).

**Table 8:** Multivariate adjusted anthropometric measures and blood pressure for men and women aged 19–64 y across processed red meat (PRM) tertiles

Biomarkers	Tertiles of processed red meat (PRM) consumption, g/d							
	Men				Women			
	T1 (0–20)	T2 (21–57)	T3 (58–284)	ANCOVA *P* value*	T1 (0–7)	T2 (8–30)	T3 (31–162)	ANCOVA *P* value*
Participants (*n*)	160	160	170		160	186	173	
Height (cm)								
Base model	176 (175, 177)^a^	177 (176, 178)^b^	178 (177, 179)^b^	0.010	162 (161, 163)^a^	163 (162, 163)^a^	163 (162, 164)^a^	0.65
+ BMI	–	–	–		–	–	–	
+ Dietary factors	176 (175, 177)^a^	177 (176, 178)^b^	178 (177, 179)^b^	0.019	162 (161, 163)^a^	163 (162, 164)^a^	163 (162, 164)^a^	0.52
Weight (kg)								
Base model	81 (79, 83)^a^	84 (82, 86)^ab^	88 (85, 90)^b^	0.0001	71 (68, 73)^a^	70 (68, 72)^a^	71 (69, 73)^a^	0.94
+ BMI	-	-	-		-	-	-	
+ Dietary factors	81 (79, 83)^a^	84 (82, 86)^ab^	87 (85, 90)^b^	0.001	71 (69, 73)^a^	70 (68, 72)^a^	71 (69, 73)^a^	0.83
BMI (kg/m^2^)								
Base model	26 (26, 27)^a^	27 (26, 27)^b^	28 (27, 28)^b^	0.006	27 (26, 28)^a^	27 (26, 27)^a^	27 (26, 28)^a^	0.88
+ BMI	–	–	–		–	–	–	
+ Dietary factors	26 (26, 27)^a^	27 (26, 27)^ab^	28 (27, 28)^b^	0.007	27 (26, 28)^a^	26 (26, 27)^a^	27 (26, 28)^a^	0.64
Waist circumference (cm)								
Base model	93 (91, 94)^a^	94 (93, 96)^a^	97 (96, 99)^b^	0.002	87 (85, 89)^a^	86 (84, 88)^a^	87 (85, 89)^a^	0.82
+ BMI	94 (93, 95)^a^	95 (94, 95)^a^	95 (94, 96)^a^	0.27	86 (85, 87)^a^	86 (85, 87)^a^	87 (85, 88)^a^	0.90
+ Dietary factors	94 (94, 95)^a^	95 (94, 95)^a^	95 (94, 96)^a^	0.52	87 (86, 88)^a^	86 (85, 87)^a^	86 (85, 88)^a^	0.71
Hip circumference (cm)								
Base model	103 (102, 104)^a^	105 (104, 106)^b^	106 (105, 108)^b^	0.0001	106 (104, 108)^a^	106 (104, 107)^a^	105 (103, 107)^a^	0.58
+ BMI	104 (103, 104)^a^	105 (104, 106)^b^	105 (104 106)^b^	0.004	106 (105, 107)^ab^	106 (105, 107)^a^	105 (104, 105)^b^	0.033
+ Dietary factors	108 (103, 104)^a^	105 (104, 106)^b^	105 (104, 106)^b^	0.006	106 (105, 107)^ab^	106 (105, 107)^a^	105 (104, 106)^b^	0.039
Waist:hip ratio								
Base model	0.90 (0.89, 0.91)^a^	0.90 (0.89, 0.91)^a^	0.91 (0.90, 0.92)^a^	0.060	0.81 (0.80, 0.82)^a^	0.81 (0.80, 0.82)^a^	0.83 (0.82, 0.84)^a^	0.061
+ BMI	0.91 (0.90, 0.91)^a^	0.90 (0.89, 0.91)^a^	0.90 (0.90, 0.91)^a^	0.22	0.81 (0.81, 0.82)^a^	0.81 (0.80, 0.82)^a^	0.83 (0.82, 0.84)^a^	0.054
+ Dietary factors	0.91 (0.90, 0.92)^a^	0.90 (0.89, 0.91)^a^	0.90 (0.90, 0.91)^a^	0.14	0.82 (0.81, 0.83)^ab^	0.81 (0.80, 0.82)^a^	0.83 (0.82, 0.84)^b^	0.039
SBP (mmHg)								
Base model	128 (126, 130)^a^	128 (126, 130)^a^	129 (127, 131)^a^	0.78	117 (115, 119)^a^	118 (116, 120)^a^	117 (115, 119)^a^	0.73
+ BMI	128 (126, 130)^a^	128 (126, 130)^a^	128 (126, 130)^a^	0.88	117 (115, 119)^a^	118 (116, 120)^a^	117 (115, 119)^a^	0.65
+ Dietary factors	129 (127, 131)^a^	128 (126, 130)^a^	128 (126, 130)^a^	0.82	117 (115, 119)^a^	118 (116, 120)^a^	117 (115, 119)^a^	0.79
DBP (mmHg)								
Base model	74 (73, 76)^a^	74 (72, 75)^a^	74 (72, 76)^a^	0.93	72 (70, 73)^a^	73 (71, 74)^a^	71 (70, 73)^a^	0.45
+ BMI	74 (73, 76)^a^	74 (72, 75)^a^	74 (72, 75)^a^	0.78	72 (70, 73)^a^	73 (71, 74)^a^	71 (70, 73)^a^	0.35
+ Dietary factors	74 (73, 76)^a^	74 (72, 75)^a^	74 (72, 75)^a^	0.69	72 (70, 73)^a^	73 (71, 74)^a^	71 (70, 73)^a^	0.39
Pulse pressure (mmHg)								
Base model	68 (66, 69)^a^	67 (65, 69)^a^	69 (67, 71)^a^	0.23	70 (69, 72)^a^	72 (70, 73)^ab^	73 (71, 75)^b^	0.032
+ BMI	68 (67, 70)^a^	67 (66, 69)^a^	69 (67, 70)^a^	0.36	70 (69, 72)^a^	72 (70, 73)^ab^	73 (71, 75)^b^	0.032
+ Dietary factors	68 (67, 70)^a^	67 (66, 69)^a^	68 (67, 70)^a^	0.55	70 (68, 71)^a^	72 (71, 73)^ab^	73 (71, 75)^b^	0.022

Values are multivariate adjusted means (95% CIs)

*Significant differences between means across processed red meat (PRM) tertiles were determined by ANCOVA controlling for age, energy intake, socioeconomic classification (SEC) and number of daily cigarettes (Base model), with additional adjustment for BMI (+ BMI) and further adjustment for fruit and vegetables, fibre, dairy, oily fish and nuts (+ dietary factors). For height and weight model 3 did not include adjustment for BMI. Tertiles that do not share a superscripts letter were significantly different at *P*<0.05 based on Bonferroni post hoc pairwise comparisons

In women, there was a significant difference in hip circumference across tertiles of PRM consumption (ANCOVA model 2: *P* = 0.033 and model 3: *P* = 0.026) with women in the highest tertile (T3) having significantly smaller hip circumference compared with women in the middle tertile (T2) of PRM consumption (model 2: T3 vs. T2 *P* = 0.043 and model 3: T3 vs. T2 *P* = 0.040, Table 8). Waist-to-hip ratio was significantly different across PRM tertiles in model 3 (ANCOVA *P* = 0.039) with women in the highest tertile (T3) having significantly larger hip-to-waist ratio compared to women in the middle (T2) tertile of PRM consumption (T3 vs. T2 *P* = 0.033). Pulse pressure was significantly different across PRM tertiles in all models (ANCOVA model 1: *P* = 0.032, model 2: *P* = 0.032 and model 3: *P* = 0.022) with women in the highest tertile (T3) of PRM having significantly higher pulse pressure compared with the lowest (T1) tertile of PRM consumption (model 3: T3 vs. T1 *P* = 0.021).

## Discussion

In this study, mean TRPRM intake was 84 g/d in men and 56 g/d in women with 57% of the total population (43% men and 69% of women) adhering to the recommendation of ≤ 70 g TRPRM per day [1]. Dietary intakes are lower than the reported TRPRM intakes in Ireland (134 g/d for men and 89 g/d for women) [21] and other European countries such as Italy (91 g/d for men and 58 g/d for women), Germany (136 g/d for men and 72 g/d for women), the Netherlands (142 g/d for men and 79 g/d for women), Spain (130 g/d for men and 67 g/d for women), Denmark (121 g/d for men and 80 g/d for women) and Sweden (89 g/d for men and 77 g/d for women), but are higher than those reported in Greece (54 g/d for men and 30 g/d for women) [22]. However, it is important to note that most of these values were obtained from EPIC data using mainly FFQ, which may not provide valid estimates of absolute intakes. In addition, there are slight differences in the definition of RM, particularly what is classed as PRM between studies, and this highlights a need for a universal definition of TRPRM [21].

RM is a source of SFA in the UK diet [15]. The balance of evidence shows that SFA consumption significantly increases the plasma concentration of LDL-C concentration, thereby increasing the risk of CHD [23], and reducing SFA intake lower CVD events [24]. However, the associations between SFA and CVD risk may also depend on food matrix, food specific fatty acids or other nutrients within SFA-rich foods. For example, results from the 10-year Multi-Ethnic Study of Atherosclerosis (MESA) showed a higher intake of SFA from meat was associated with greater CVD risk, whereas dairy SFA was related to lower CVD risk [25]. Indeed, when 2% of energy from meat SFA was replaced by 2% energy from dairy SFA, risk of CVD was 25% lower (HR 95% CI 0.75; 0.63, 0.91) [25]. We reported that the mean SFA intakes were above the 11% (of food energy) recommendation for both men and women and intakes were significantly higher with increasing RM and PRM intake. We did not find any significant differences in LDL-C across RM tertiles. However, we did find significantly higher LDL-C in women but not men with the highest consumption of PRM, although it is important to note that this study was a cross-sectional study. Furthermore, in our analysis, the health biomarker estimates did not change significantly following adjustment for dietary factors (fruit and vegetables, fibre, dairy, oily fish and nuts), suggesting that the wider dietary pattern had minimal impact on the observed effects.

We found the percentage of men below the LRNI for zinc was decreased with increasing tertiles of RM and PRM consumption, particularly for RM. Zinc is required for the activity of many different enzymes in the body, which are involved in major metabolic pathways. Therefore, zinc is needed for a wide range of biochemical, immunological and clinical functions. Consequently, zinc deficiency affects a number of different functions in the body including physical growth, immune competency and neuro-behavioural development and reproductive function [26]. Indeed studies have shown that fertile men have higher semen zinc levels compared with their infertile counterparts [27]. We also observed the percentage of women below the LRNI for iron was decreased with increasing tertiles of RM. Iron is involved in a number of important metabolic processes in the body, including oxygen transport, DNA synthesis and electron transport. Studies have also associated iron deficiency with reduced cognitive function, mental health and heightened fatigue [28]. A recent secondary analysis of cross-sectional data from the 2011 Food Consumption Survey showed dietary patterns with the highest intakes of processed red meat was associated with a lower Alternative Healthy Eating Index score but there were no significant differences in dietary intakes of zinc and iron across dietary patterns [21].

We observed higher PRM was associated with significantly higher Hb A_1c_ concentration in women. In a cross-sectional analysis of 3690 diabetes-free women from the Nurses Health Study, Lay and colleagues also found total, unprocessed and processed RM intakes to be associated with higher Hb A_1c_ and plasma insulin concentrations [29]. However, the authors suggested that BMI accounted for a significant proportion of the associations with Hb A_1c_ and plasma insulin concentrations. We did not find this to be the case in our analysis.

We also found higher PRM was associated with significantly increased body weight and BMI in men. These findings are in line with a cross-sectional analysis of 1999–2004 NHANES data that showed positive correlations between meat consumption (all animal source foods), other meat products (frankfurter, sausages, organ meats, food mixtures containing meat, poultry and fish) and higher BMI and waist circumference [30]. In addition, a more recent analysis of 2005–2010 NHANES data suggests that adiposity, particularly accumulation of abdominal fat, accounts for a significant proportion of the associations between RM consumption and insulin resistance and inflammation [31].

To date, results from randomized controlled trials (RCTs) on the effect of RM consumption on CVD risk factors are inconsistent [9–11]. However, it has been suggested that these inconsistencies may be partly due to the composition of the comparison diet [11]. For example, in their meta-analysis of 36 RCTs, Guasch-Ferre and colleagues showed that relative to all comparison diets combined, RM consumption had no differential effects on TC, LDL-C, HDL-C apolipoproteins A1 and B or blood pressure, but resulted in lesser decrease in triacylglycerol concentrations. However, when the analysis was stratified by type of comparison diet (usual diet, high-quality protein foods, carbohydrate foods, fish or poultry), substituting RM for high-quality plant foods showed more favorable changes in total and LDL-C [11].

In addition, Lenighan et al. [21] found there were no associations between dietary patterns containing varying levels of RM and unprocessed RM and risk factors for CVD and type 2 diabetes [21]. A possible explanation for the difference in findings between studies may be due to differences in RM and unprocessed RM intakes. For example, in the study by Lenighan et al. [21], average intakes of RM and unprocessed RM were lower (RM 57.1 g/d and unprocessed RM 86.2 g/d) in the high meat dietary pattern compared to intakes in our high RM and PRM tertiles (RM: 81.9 and PRM: 90.4). On the other hand, our findings are in line with the most recent meta-analysis, which showed a significant positive relationship with processed meat intake and CHD, with a 50 g/day serving resulting in a significantly higher risk of CHD (relative risk 1.42; 95% CI 1.07, 1.89, *P* = 0.04) compared with a 100 g serving of RM (relative risk 1.00; 95% CI 0.81, 1.23, *P* = 0.36) [5]. Moreover, in a multivariable case–cohort analysis using data from 120,852 participants in The Netherlands Cohort Study unprocessed red meat intake was not associated with overall and cause-specific mortality [32]. However, processed meat was significantly positively associated with overall (HR Q5 Vs. Q1; 1.21 95% CI 1.02, 1.44) and cardiovascular mortality (HR Q5 Vs. Q1; 1.26 95% CI 1.01, 1.26) [32]. These associations became nonsignificant when an adjustment for nitrite intake was made, suggesting nitrite intake was a key driver of these associations [32]. In a meta-analysis, both RM and PRM were associated with incident diabetes, however, the association was less strong with unprocessed RM [5]. Other studies have also found associations with RM and/or PRM with type 2 diabetes. Furthermore, findings from the EPIC-InterAct prospective case–cohort study showed significant positive associations with incident type 2 diabetes with increasing consumption of total meat (HR 1.08; 95% CI 1.05, 1.12), RM (HR 1.08; 95% CI 1.03, 1.13) and processed meat (HR 1.12; 95% CI 1.05, 1.19) [33]. A meta-analysis by Pan et al. [34], which included 442,101 participants showed that consumption of both unprocessed RM and PRM was significantly associated with risk of type 2 diabetes. In this study, intakes of RM and haem iron were strongly correlated, and when adjustment was made for haem iron intake the association between RM and type 2 diabetes risk was lost [34]. This suggests that the haem iron component of RM may be linked with the increased risk of type 2 diabetes with RM consumption, but the exact mechanism of action has not yet been identified. There is some evidence to suggest that iron increases the production of reactive oxygen species, which may then damage the insulin-producing pancreatic cells [35], but further studies are needed to fully determine this.

We also observed significantly higher sodium intakes in both men and women with increasing tertiles of PRM consumption, but not with RM tertiles. Indeed, Micha et al. highlighted that PRM contains approximately 400% more sodium than RM per gram [5]. The consumption of high levels of sodium has been associated with increased risk of hypertension, which is a key risk factor for CVD [36]. Therefore, the higher levels of sodium in women with the highest intakes of PRM could contribute to the higher pulse pressure observed in the women in this study, although further research would be required to confirm this. It is also important to bear in mind that the sodium levels in the NDNS may underestimate total sodium intake from the diet as they include only sodium from food and do not include additional salt added in cooking or at the table by participants.

In our study, the PRM food group included sausages, bacon and ham and other PRM (such as corned beef and salami), with sausages, bacon and ham making up the vast majority of processed meat consumed. PRM such as bacon and ham often contain salt enriched with nitrates or nitrites to improve preservation. Meat containing nitrate and nitrite may lead to the formation of N-nitroso compounds (NOC), which have been suggested to contribute towards the pathogenesis of T2D [37]. However, more studies are needed to further identify the mechanisms of action.

We also observed significantly higher *trans*-fatty acids (TFA) intake with increasing RM consumption for both men and women, but not with diets with higher PRM. High intakes of TFAs are associated with an increased risk of CVD [38]. However, it is important to note that average TFA intakes of adults in the NDNS were below recommendations (2% food energy). The differences in TFAs between RM and PRM seen in our study is likely due to enforced or voluntary changes in the refining and processing of plant oils and vegetable fats, which have led to significantly less industrially produced TFAs (iTFAs) in the food chain. Indeed, data from the most recent NDNS years 7 and 8 (2014/15–2015/16) show that average UK intakes are below recommendations of 2% of food energy for all ages/sex [13]. The reduction in iTFAs has led to an increase in the relative contribution of ruminant TFA (rTFA) to total TFA intakes. Although there are strong data on the association between iTFA consumption and CVD mortality [39], there are insufficient data linking rTFAs with CVD.

We observed higher carbohydrate and starch intakes with increasing tertiles for RM and PRM. It is likely that this is a reflection of the wider diet of the individuals in our analysis, rather than as a direct result of RM and PRM intakes. This association may also reflect the way RM and PRM is consumed, for example with starchy foods such as potatoes. However, to fully determine this, a detailed food group analysis would need to be conducted.

This analysis has a number of strengths, for example the NDNS is designed to be representative of the UK population. In addition, the analysis was based on disaggregated red meat and processed red meat intake data, which is more meaningful. However, a limitation of this analysis is the cross-sectional nature of the NDNS, which means observed associations do not imply causation. In addition, it is important to highlight that the associations we have observed between RM and PRM intake may not be a direct result of RM and PRM consumption but may also be due to residual confounding by other aspects of the diets of the participants in the study. Furthermore, dietary intakes were self-reported, thus an element of reporting bias may be present. It may also be the case that the 4 day diet diary did not capture habitual intake in some participants. In addition, some participants may have changed their diet since dietary assessment and nurse visit, which may have impacted on the proximity of the dietary assessment to blood samples. It is also important to highlight that, there was a 2–4-month period between dietary assessment and nurse visit [15]. This may have resulted in the cardio-metabolic risk markers not being a true reflection of the participants’ dietary intake at that time.

There was also a large number of missing blood samples, therefore the biomarkers of health analysis may not represent the total study population. We did not adjust for multiple testing in our statistical models due to the many comparisons that were performed in each analysis, the *P* values should, therefore, be interpreted with caution. Moreover, there may be a differential effect of processing method and meat species such as beef, lamb and pork on cardio-metabolic risk factors, but the current analysis did not have sufficient power to detect these differences.

## Conclusions

In conclusion, 57% men and 31% of women had total red and processed meat above the recommendation of ≤ 70 g total red and processed meat per day [1]. Some current diets in the UK containing lower RM (< 13 g/day) may have implications for sub-optimal micro-nutrient intakes, particularly for iron and zinc. We found higher PRM consumption was associated with significantly higher BMI and hip circumference in men and higher TC, LDL-C, Hb A_1c_ and PP in women, which was not observed for higher RM consumption. These data need confirmation, but support dietary guidance for reduction in PRM consumption.

## Supplementary Information

Below is the link to the electronic supplementary material.

Supplementary file1 (DOCX 21 KB)

## Abbreviations

ANOVA: Analysis of variance
BMI: Body mass index
CVD: Cardiovascular disease
CHD: Coronary heart disease
HEI: Healthy eating index
HDL-C: High-density lipoprotein cholesterol
HbA1c: Glycated haemoglobin
LRNI: Lower reference nutrient intake
LDL-C: Low-density lipoprotein cholesterol
MUFA: Monounsaturated fatty acids
NDNS: National Diet and Nutrition Survey
NHANES: National Health and Nutrition Examination Survey
PP: Pulse pressure
PRM: Processed red meat
RM: Red meat
SD: Standard deviation
TAG: Triacylglycerol
TC: Total cholesterol
TRPRM: Total red and processed red meat
TFA: *trans*-Fatty acids
