# Supplementary material for: Associations between red meat, processed red meat and total red and processed red meat consumption, nutritional adequacy and markers of health and cardio-metabolic diseases in British adults: a cross-sectional analysis using data from UK National Diet and Nutrition Survey
Source: Eur J Nutr. 2021 Feb 7;60(6):2979–97. doi: 10.1007/s00394-021-02486-3 (PMC8354925; doi:10.1007/s00394-021-02486-3)
Supplement: Supplementary file 1 — Supplementary file1 (DOCX 21 KB) [file 394_2021_2486_MOESM1_ESM.docx]

**Supplemental table 1** Multivariate adjusted daily intakes of macro- and micro-nutrients and percentage of men and women below the Lower recommended Nutrient Intakes (LRNI) across total red and processed red meat (TRPRM) tertiles

|  | Tertiles of total red and processed red meat (TRPRM) consumption, g/d | | | | | | | | |
| --- | --- | --- | --- | --- | --- | --- | --- | --- | --- |
|  | Men | | | |  | Women | | | |
|  | T1 | T2 | T3 | ANCOVA |  | T1 | T2 | T3 | ANCOVA |
|  | (0-55) | (56-103) | (104-344) | *P*-value^1^ |  | (0-31) | (32-67) | (68-233) | *P*-value^1^ |
| Participants (*n*) | 266 | 268 | 267 |  |  | 321 | 321 | 315 |  |
| Total energy (MJ) | 8.1 (7.8, 8.4)^a^ | 8.8 (8.5, 9.1)^b^ | 10.1 (9.8, 10.4)^c^ | 0.0001 |  | 6.1 (5.9, 6.3)^a^ | 6.8 (6.6, 7.0)^b^ | 7.2 (7.0, 7.4)^c^ | 0.0001 |
| Protein (g) | 82 (79, 85)^a^ | 84 (81, 87)^a^ | 94 (91, 97)^b^ | 0.0001 |  | 60 (58, 61)^a^ | 64 (63, 65)^b^ | 72 (71, 73)^c^ | 0.0001 |
| Fat (g) | 77 (75, 79)^a^ | 79 (77, 81)^ab^ | 81 (79, 83)^b^ | 0.004 |  | 58 (57, 59)^a^ | 59 (57, 60)^a^ | 61 (60, 62)^b^ | 0.003 |
| % food energy | 33 (33, 34)^a^ | 36 (35, 36)^b^ | 36 (35, 37)^b^ | 0.0001 |  | 33 (33, 34)^a^ | 34 (33, 35)^a^ | 35 (35, 36)^b^ | 0.0001 |
| SFA (g) | 28 (27, 29)^a^ | 29 (28, 30)^ab^ | 30 (29, 31)^b^ | 0.017 |  | 21 (20, 22)^a^ | 21 (20, 22)^a^ | 23 (22, 23)^b^ | 0.0001 |
| % food energy | 12 (12, 12)^a^ | 13 (13, 13)^b^ | 13 (13, 14)^b^ | 0.0001 |  | 12 (12, 12)^a^ | 12 (12, 13)^a^ | 13 (13, 13)^b^ | 0.0001 |
| Cis MUFA (g) | 28 (27, 28)^a^ | 29 (28, 30)^b^ | 30 (29, 31)^b^ | 0.0001 |  | 21 (20, 21)^a^ | 21 (21, 22)^ab^ | 22 (21, 22)^b^ | 0.005 |
| % food energy | 12 (12, 12)^a^ | 13 (13, 14)^b^ | 13 (13, 14)^b^ | 0.0001 |  | 12 (12, 12)^a^ | 12 (12, 13)^b^ | 13 (12, 13)^b^ | 0.0001 |
| Cis n-6 FAs (g) | 12 (11, 12)^a^ | 11 (11, 12)^a^ | 12 (11, 12)^a^ | 0.35 |  | 8.8 (8.4, 9.1)^a^ | 8.7 (8.4, 9.1)^a^ | 8.6 (8.3, 8.9)^a^ | 0.80 |
| % food energy | 5.2 (5, 5.4)^a^ | 5.2 (5, 5.4)^a^ | 5.1 (4.9, 5.3)^a^ | 0.76 |  | 5.1 (4.9, 5.3)^a^ | 5.1 (4.9, 5.2)^a^ | 5.0 (4.9, 5.2)^a^ | 0.96 |
| Cis n-3 FAs (g) | 2.2 (2.1, 2.3)^a^ | 2.3 (2.2, 2.4)^a^ | 2.1 (2, 2.2)^a^ | 0.11 |  | 2.0 (1.9, 2.1)^a^ | 1.8 (1.7, 1.9)^b^ | 1.6 (1.5, 1.7)^c^ | 0.0001 |
| % food energy | 1.0 (0.9, 1.0)^a^ | 1.0 (1.0, 1.1)^a^ | 0.9 (0.9, 1.0)^b^ | 0.02 |  | 1.1 (1.1, 1.2)^a^ | 1.1 (1, 1.1)^a^ | 0.9 (0.9, 1.0)^b^ | 0.0001 |
| TFAs (g) | 1.4 (1.3, 1.4)^a^ | 1.5 (1.4, 1.6)^ab^ | 1.6 (1.5, 1.7)^b^ | 0.001 |  | 1.0 (1.0, 1.1)^a^ | 1.1 (1, 1.1)^b^ | 1.2 (1.2, 1.3)^c^ | 0.0001 |
| % food energy | 0.60 (0.56, 0.63)^a^ | 0.67 (0.64, 0.71)^b^ | 0.70 (0.67, 0.74)^b^ | 0.0001 |  | 0.59 (0.56, 0.63)^a^ | 0.62 (0.59, 0.65)^a^ | 0.72 (0.69, 0.75)^b^ | 0.0001 |
| Carbohydrate (g) | 266 (262, 271)^a^ | 246 (242, 251)^b^ | 242 (237, 247)^b^ | 0.0001 |  | 206 (202, 209)^a^ | 196 (193, 199)^b^ | 185 (182, 188)^c^ | 0.0001 |
| % food energy | 50 (49, 51)^a^ | 47 (46, 48)^b^ | 45 (44, 46)^c^ | 0.0001 |  | 51 (50, 51)^a^ | 48 (48, 49)^b^ | 46 (45, 47)^c^ | 0.0001 |
| Total sugars (g) | 111 (107, 115)^a^ | 105 (101, 110)^b^ | 97 (93, 101)^c^ | 0.0001 |  | 84 (81, 87)^a^ | 87 (84, 89)^a^ | 76 (73, 79)^b^ | 0.0001 |
| % food energy | 21 (20, 22)^a^ | 20 (19, 21)^a^ | 18 (17, 19)^b^ | 0.0001 |  | 21 (20, 22)^a^ | 21 (20, 22)^a^ | 19 (18, 20)^b^ | 0.0001 |
| Englyst fibre (g) | 16 (15, 16)^a^ | 14 (14, 15)^b^ | 14 (14, 15)^b^ | 0.0001 |  | 14 (13, 14)^a^ | 12 (12, 13)^b^ | 12 (12, 12)^b^ | 0.0001 |
| Vitamin A (µg)^2^ | 1031 (923, 1140)^a^ | 969 (854, 1084)^ab^ | 814 (696, 933)^b^ | 0.033 |  | 1022 (911, 1133)^a^ | 888 (784, 993)^a^ | 990 (884, 1096)^a^ | 0.19 |
| % below LRNI | 9 | 12 | 10 |  |  | 7 | 7 | 5 |  |
| Thiamin (mg) | 1.5 (1.5, 1.6)^a^ | 1.5 (1.5, 1.6)^a^ | 1.8 (1.7, 1.8)^b^ | 0.0001 |  | 1.2 (1.2, 1.3)^a^ | 1.2 (1.2, 1.3)^a^ | 1.4 (1.3, 1.4)^b^ | 0.0001 |
| % below LRNI | 0 | 0 | 0 |  |  | 0 | 0 | 0 |  |
| Riboflavin (mg) | 1.8 (1.7, 1.9)^a^ | 1.7 (1.6, 1.8)^a^ | 1.7 (1.7, 1.8)^a^ | 0.15 |  | 1.3 (1.3, 1.4)^a^ | 1.3 (1.3, 1.4)^a^ | 1.4 (1.3, 1.4)^b^ | 0.45 |
| % below LRNI | 6 | 4 | 2 |  |  | 16 | 12 | 8 |  |
| Niacin equivalent (mg) | 41 (40, 43)^a^ | 42 (41, 44)^ab^ | 45 (43, 46)^b^ | 0.029 |  | 30 (29, 31)^a^ | 32 (31, 33)^b^ | 33 (32, 34)^b^ | 0.0001 |
| % below LRNI | 0 | 0 | 0 |  |  | 0 | 0 | 0 |  |
| Vitamin B6 (mg) | 2.5 (2.3, 2.6)^a^ | 2.6 (2.5, 2.7)^a^ | 2.6 (2.5, 2.8)^a^ | 0.052 |  | 1.8 (1.7, 1.9)^a^ | 1.9 (1.8, 2.0)^a^ | 1.9 (1.9, 2.0)^a^ | 0.08 |
| % below LRNI | 4 | 3 | 1 |  |  | 18 | 10 | 3 |  |
| Vitamin B12 (µg) | 5.4 (4.9, 5.8)^a^ | 5.7 (5.2, 6.1)^a^ | 6.1 (5.6, 6.6)^a^ | 0.12 |  | 4.4 (4.0, 4.7)^a^ | 4.5 (4.2, 4.8)^a^ | 4.8 (4.5, 5.1)^a^ | 0.20 |
| % below LRNI | 2 | 1 | 0 |  |  | 5 | 0 | 0 |  |
| Folate (µg) | 292 (280, 304)^a^ | 294 (282, 307)^a^ | 278 (265, 290)^a^ | 0.15 |  | 240 (232, 249)^a^ | 223 (216, 231)^b^ | 224 (216, 232)^b^ | 0.007 |
| % below LRNI | 1 | 1 | 1 |  |  | 7 | 4 | 2 |  |
| Vitamin C (mg) | 92 (85, 99)^a^ | 82 (75, 90)^ab^ | 75 (67, 83)^b^ | 0.008 |  | 86 (80, 93)^a^ | 86 (80, 92)^a^ | 74 (68, 80)^b^ | 0.005 |
| % below LRNI | 2 | 2 | 0 |  |  | 1 | 1 | 1 |  |
| Sodium (mg) | 2464 (2386, 2543)^a^ | 2590 (2507, 2673)^b^ | 2808 (2723, 2893)^c^ | 0.0001 |  | 1920 (1863, 1977)^a^ | 1936 (1882, 1990)^a^ | 2136 (2081, 2191)^b^ | 0.0001 |
| % below LRNI | 1 | 0 | 1 |  |  | 2 | 0 | 0 |  |
| Potassium (mg) | 3038 (2968, 3109)^a^ | 2999 (2925, 3074)^a^ | 3054 (2977, 3131)^a^ | 0.58 |  | 2512 (2456, 2569)^a^ | 2486 (2433, 2540)^a^ | 2530 (2476, 2585)^a^ | 0.52 |
| % below LRNI | 15 | 12 | 4 |  |  | 31 | 24 | 16 |  |
| Calcium (mg) | 938 (909, 967)^a^ | 866 (836, 897)^b^ | 850 (819, 882)^b^ | 0.0001 |  | 741 (719, 764)^a^ | 699 (678, 720)^b^ | 689 (668, 711)^b^ | 0.003 |
| % below LRNI | 5 | 7 | 3 |  |  | 10 | 8 | 8 |  |
| Magnesium (mg) | 293 (287, 300)^a^ | 279 (272, 286)^b^ | 273 (266, 281)^b^ | 0.0001 |  | 235 (230, 240)^a^ | 220 (216, 225)^b^ | 216 (212, 221)^b^ | 0.0001 |
| % below LRNI | 18 | 19 | 8 |  |  | 18 | 13 | 8 |  |
| Iron (mg) | 12 (11, 12)^a^ | 12 (11, 12)^a^ | 12 (12, 12)^a^ | 0.34 |  | 10 (9.0, 10)^a^ | 9.0 (9.0, 10)^a^ | 10 (9.0, 10)^a^ | 0.21 |
| % below LRNI | 3 | 1 | 0 |  |  | 33 | 27 | 18 |  |
| Haem iron (mg) | 0.48 (0.43, 0.53)^a^ | 0.82 (0.76, 0.87)^b^ | 1.18 (1.13, 1.24)^c^ | 0.0001 |  | 0.34 (0.30, 0.38)^a^ | 0.57 (0.53, 0.60)^b^ | 0.85 (0.81, 0.88)^c^ | 0.0001 |
| Non-haem iron (mg) | 11 (11, 12)^a^ | 11 (10, 11)^a^ | 11 (10, 11)^a^ | 0.21 |  | 9.2 (9.0, 9.4)^a^ | 8.9 (8.6, 9.1)^b^ | 8.9 (8.6, 9.1)^b^ | 0.076 |
| Zinc (mg) | 8.6 (8.4, 8.9)^a^ | 9.4 (9.1, 9.7)^b^ | 11 (11, 12)^c^ | 0.0001 |  | 6.6 (6.4, 6.8)^a^ | 7.2 (7.1, 7.4)^b^ | 8.8 (8.6, 9.0)^c^ | 0.0001 |
| % below LRNI | 16 | 7 | 0 |  |  | 12 | 2 | 1 |  |
| Iodine (µg) | 188 (180, 195)^a^ | 179 (170, 187)^ab^ | 173 (164, 181)^b^ | 0.038 |  | 142 (136, 147)^a^ | 136 (130, 141)^ab^ | 132 (127, 137)^b^ | 0.052 |
| % below LRNI | 7 | 6 | 1 |  |  | 12 | 11 | 8 |  |
| Selenium (µg) | 54 (52, 56)^a^ | 54 (51, 56)^a^ | 55 (52, 58)^a^ | 0.82 |  | 44 (42, 46)^a^ | 43 (42, 45)^a^ | 43 (41, 45)^a^ | 0.69 |
| % below LRNI | 34 | 30 | 14 |  |  | 60 | 55 | 44 |  |

Values are multivariate adjusted means (95% CIs) or percentages unless otherwise stated.

^1^Significant differences between total red and processed red meat (TRPRM) tertiles were determined by ANCOVA controlling for age, energy intake, socioeconomic classification (SEC) and number of daily cigarettes. Tertiles that do not share a superscripts letter were significantly different at the 5% level based on Bonferroni post hoc pairwise comparisons.^2^Retinol equivalents.
